# Optimal and typical $L^2$ discrepancy of 2-dimensional lattices

**DOI:** 10.1007/s10231-024-01440-4

**Published:** 2024-03-23

**Authors:** Bence Borda

**Affiliations:** Graz University of Technology, Steyrergasse 30, 8010 Graz, Austria

**Keywords:** Continued fraction, Quadratic irrational, Korobov lattice, Symmetrization, Low discrepancy, Limit distribution, 11K38, 11J83

## Abstract

We undertake a detailed study of the $L^2$ discrepancy of 2-dimensional Korobov lattices and their irrational analogues, either with or without symmetrization. We give a full characterization of such lattices with optimal $L^2$ discrepancy in terms of the continued fraction partial quotients, and compute the precise asymptotics whenever the continued fraction expansion is explicitly known, such as for quadratic irrationals or Euler’s number *e*. In the metric theory, we find the asymptotics of the $L^2$ discrepancy for almost every irrational, and the limit distribution for randomly chosen rational and irrational lattices.

## Introduction

The extreme discrepancy of a finite point set $P \subset [0,1)^d$ in the unit cube is defined as

$$\begin{aligned} D_{\textrm{extr}} (P) = \sup _{A \subseteq [0,1)^d} \left| |P \cap A| - |P| \lambda (A) \right| , \end{aligned}$$

where the supremum is over all axis-parallel boxes $A=[a_1, b_1) \times \cdots \times [a_d, b_d) \subseteq [0,1)^d$, and $\lambda$ is the Lebesgue measure. The $L^2$ discrepancy is defined as the $L^2$ average over all axis-parallel boxes anchored at the origin:

$$\begin{aligned} D_2 (P) = \left( \int _{[0,1]^d} \left( |P \cap [0,x_1) \times \cdots \times [0,x_d) | -|P|x_1 \cdots x_d \right) ^2 \, \textrm{d} x_1 \cdots \textrm{d} x_d \right) ^{1/2}. \end{aligned}$$

Clearly, $D_2 (P) \le D_{\textrm{extr}}(P)$. The extreme and the $L^2$ discrepancy are common measures of equidistribution, with direct applications to numerical integration; for a general introduction we refer to the monograph Drmota–Tichy [16]. In dimension $d=2$, a seminal result of K. Roth [26] states that every finite point set $P \subset [0,1)^2$ satisfies $D_2(P) \gg \sqrt{\log |P|}$ with a universal implied constant. This is known to be sharp, with several explicit constructions e.g. based on digital nets attaining the optimal order $D_2(P) \ll \sqrt{\log |P|}$, see [15]. The corresponding result for the extreme discrepancy due to W. Schmidt [16, p. 44] states that for every finite point set $P \subset [0,1)^2$, we have $D_{\textrm{extr}} (P) \gg \log |P|$ with a universal implied constant, and this is again sharp with several explicit constructions attaining the optimal order $D_{\textrm{extr}} (P) \ll \log |P|$. In contrast, in dimensions $d \ge 3$ the best known constructions of finite point sets $P \subset [0,1)^d$ satisfy $D_{\textrm{extr}} (P) \ll (\log |P|)^{d-1}$, but the optimal order remains an important open problem.

Given a positive integer *N* and integers $p_1, \ldots , p_d$, the set

$$\begin{aligned} \mathcal {L}_{p_1, \ldots , p_d,N} = \left\{ \left( \left\{ \frac{np_1}{N} \right\} , \ldots , \left\{ \frac{np_d}{N} \right\} \right) \in [0,1)^d \,: \, 0 \le n \le N-1 \right\} , \end{aligned}$$

where $$\{ \cdot \}$$ denotes the fractional part function, is called a *rank-1 lattice rule* or a *Korobov lattice*. The terminology is explained by the fact that $$\mathcal {L}_{p_1,\ldots , p_d,N}$$ is the intersection of the unit cube $$[0,1)^d$$ and the dual of the lattice^1^$$\Gamma = \{ (n_1,\ldots , n_d) \in \mathbb {Z}^d \,: \, n_1 p_1 + \cdots + n_d p_d \equiv 0 \pmod {N} \}$$. Korobov lattices have been extensively used as quadrature rules in numerical integration. Finding lattice points $$(p_1,\ldots , p_d) \in \mathbb {Z}^d$$ that make the approximation error

$$\begin{aligned} \int _{[0,1]^d} f(x) \, \textrm{d}x \approx \frac{1}{N} \sum _{n=0}^{N-1} f \left( \left\{ \frac{np_1}{N} \right\} , \ldots , \left\{ \frac{np_d}{N} \right\} \right) \end{aligned}$$

suitably small for given classes of integrable functions *f* is known as the method of “good lattice points”. We refer to the monograph of Dick, Kritzer and Pillichshammer [14] for a comprehensive survey and a history of lattice rules.

Bykovskii [11] showed that in any dimension $d \ge 3$, for any integer $N \ge 3$ there exist integers $p_1, \ldots , p_d$, each coprime with *N*, such that $D_{\textrm{extr}}(\mathcal {L}_{p_1, \ldots , p_d,N}) \ll (\log N)^{d-1} \log \log N$. Note that this matches the best known constructions up to a factor of $\log \log N$. The proof is probabilistic, see also [19, 20]. We refer to [14, Chapter 5] for an algorithm that produces a good lattice point such that $D_{\textrm{extr}}(\mathcal {L}_{p_1, \ldots , p_d,N}) \ll (\log N)^d$, and for results on the discrepancy with respect to all convex sets.

Dimension $d=2$ is rather special. In this case, given a positive integer *N*, and integers $p_1, p_2$, both coprime with *N*, we have $\mathcal {L}_{p_1,p_2,N} = \mathcal {L}_{p,1,N}$ with $p=p_2^* p_1$, where $p_2^*$ is the multiplicative inverse of $p_2 \pmod {N}$. In particular, Korobov lattices are parametrized by a single rational number *p*/*N*, and their distribution properties can be characterized in terms of the continued fraction expansion of *p*/*N*. The main goal of this paper is to study the $L^2$ discrepancy of 2-dimensional Korobov lattices, and their analogues corresponding to irrational values of the parameter.

More precisely, given $\alpha \in \mathbb {R}$ and $N \in \mathbb {N}$, we will consider the *N*-element set

$$\begin{aligned} L(\alpha , N) = \left\{ \left( \{ n \alpha \}, \frac{n}{N} \right) \in [0,1)^2 \,: \, 0 \le n \le N-1 \right\} , \end{aligned}$$

and the 2*N*-element set

$$\begin{aligned} S(\alpha , N) = \left\{ \left( \{ \pm n \alpha \}, \frac{n}{N} \right) \in [0,1)^2 \,: \, 0 \le n \le N-1 \right\} . \end{aligned}$$

Note that $$L(\alpha , N)$$ is the intersection of the unit square $$[0,1)^2$$ and the lattice spanned by the vectors $$(\alpha , 1/N)$$ and (1, 0). We call $$S(\alpha , N)$$ the symmetrization of $$L(\alpha , N)$$; more precisely, $$S(\alpha , N)$$ is the union of $$L(\alpha , N)$$ and its reflection about the vertical line $$x=1/2$$. We study both rational and irrational values of $$\alpha $$.

The equidistribution properties of $S(\alpha , N)$ and $L(\alpha , N)$, in particular their $L^2$ discrepancy, are closely related to the Diophantine approximation properties of $\alpha$. Throughout this paper, $\alpha =[a_0;a_1,a_2,\dots ]$ will denote the (finite or infinite) continued fraction expansion of $\alpha$, and $p_k/q_k=[a_0;a_1,\dots , a_k]$ its convergents. In the rational case it will not matter which of the two possible expansions is chosen. Roughly speaking, we will show that for $N \approx q_K$,

$$\begin{aligned} D_2^2 (S(\alpha , N)) \approx \sum _{k=1}^K a_k^2 \quad \text {and} \quad D_2^2(L(\alpha , N)) \approx \sum _{k=1}^K a_k^2 + \left( \sum _{k=1}^K (-1)^k a_k \right) ^2. \end{aligned}$$

See Propositions 7 and 8 below for a precise formulation.

Our first result characterizes all irrationals for which $S(\alpha , q_K)$ resp. $L(\alpha , q_K)$ attains optimal $L^2$ discrepancy as $K \rightarrow \infty$. We also consider the same problem for $S(\alpha , N)$ and $L(\alpha , N)$ as $N \rightarrow \infty$. The first equivalence below generalizes a result of Davenport [12], who showed that $S(\alpha , N)$ attains optimal $L^2$ discrepancy whenever $\alpha$ is badly approximable, i.e. $a_k \ll 1$.

### Theorem 1

Let $\alpha =[a_0;a_1,a_2, \dots ]$ be irrational. We have

$$\begin{aligned} \begin{aligned} D_2 (S(\alpha ,N)) \ll \sqrt{\log N} \,\,&\Longleftrightarrow \,\, D_2 (S(\alpha ,q_K)) \ll \sqrt{\log q_K} \,\, \Longleftrightarrow \,\, \frac{1}{K} \sum _{k=1}^K a_k^2 \ll 1, \\ D_2 (L(\alpha ,q_K)) \ll \sqrt{\log q_K} \,\,&\Longleftrightarrow \,\, \frac{1}{K} \sum _{k=1}^K a_k^2 \ll 1 \text { and } \frac{1}{\sqrt{K}} \left| \sum _{k=1}^K (-1)^k a_k \right| \ll 1. \end{aligned} \end{aligned}$$

### Remark 1

We also give an almost complete answer for the unsymmetrized lattice $L(\alpha , N)$ with general *N*: under the assumption $a_k \ll \sqrt{k}/(\log k)^2$, we have

$$\begin{aligned} D_2 (L(\alpha ,N)) \ll \sqrt{\log N} \,\, \Longleftrightarrow \,\, \frac{1}{K} \sum _{k=1}^K a_k^2 \ll 1 \text { and } \frac{1}{\sqrt{K}} \left| \sum _{k=1}^K (-1)^k a_k \right| \ll 1. \end{aligned}$$

In the special case of a badly approximable $\alpha$, this equivalence was observed in [5, 7]. Note that $K^{-1} \sum _{k=1}^K a_k^2 \ll 1$ implies that $a_k \ll \sqrt{k}$; we do not know whether the slightly stronger extra assumption $a_k \ll \sqrt{k}/(\log k)^2$ can be removed.

More precise results can be deduced for an irrational $\alpha$ whose continued fraction expansion is explicitly known. The most interesting case is that of quadratic irrationals, whose continued fractions are of the form $\alpha =[a_0;a_1,\dots , a_r,\overline{a_{r+1}, \dots , a_{r+p}}]$, where the overline denotes the period. Note that in this case $\sum _{k=1}^K (-1)^k a_k = A(\alpha ) K +O(1)$ with some constant $A(\alpha )$. In fact, $A(\alpha )=0$ if *p* is odd, and $A(\alpha )=p^{-1} \sum _{k=1}^p (-1)^{r+k} a_{r+k}$ (possibly zero) if *p* is even. We also have $\log q_K=\Lambda (\alpha ) K+O(1)$ with some constant $\Lambda (\alpha )>0$. In fact, $\Lambda (\alpha ) = p^{-1} \log \eta$, where $\eta >1$ is the larger of the two eigenvalues of the matrix

$$\begin{aligned} \left( \begin{array}{cc} 0 &{} 1 \\ 1 &{} a_{r+1} \end{array} \right) \left( \begin{array}{cc} 0 &{} 1 \\ 1 &{} a_{r+2} \end{array} \right) \cdots \left( \begin{array}{cc} 0 &{} 1 \\ 1 &{} a_{r+p} \end{array} \right) . \end{aligned}$$

### Theorem 2

Let $\alpha$ be a quadratic irrational, and let $A(\alpha )$ and $\Lambda (\alpha )$ be as above. There exists a constant $c(\alpha )>0$ such that

$$\begin{aligned} D_2^2(S(\alpha , N)) = c(\alpha ) \log N +O(1), \end{aligned}$$

and

$$\begin{aligned} D_2^2(L(\alpha , N)) = \left\{ \begin{array}{ll} \frac{3}{2} c(\alpha ) \log N + O((\log \log N)^4) &{} \text {if } A(\alpha )=0, \\ \frac{A(\alpha )^2}{144 \Lambda (\alpha )^2} (\log N)^2 + O(\log N) &{} \text {if } A(\alpha ) \ne 0. \end{array} \right. \end{aligned}$$

The implied constants depend only on $\alpha$.

We proved the same result for $S(\alpha , N)$ with the slightly worse error term $O(\log \log N)$ in a previous paper [9]. In contrast to $A(\alpha )$ and $\Lambda (\alpha )$, there seems to be no simple way to compute the value of $c(\alpha )$ directly from the continued fraction expansion. The latter constant first appeared in certain lattice point counting problems studied in detail by Beck [1–3], who showed that it is related to the arithmetic of the ring of algebraic integers of the real quadratic field $\mathbb {Q}(\alpha )$, and computed its explicit value for any quadratic irrational; for instance,

$$\begin{aligned} c \left( \frac{1+\sqrt{5}}{2} \right) = \frac{1}{30 \sqrt{5} \log \frac{1+\sqrt{5}}{2}} \quad \text {and} \quad c(\sqrt{3}) = \frac{1}{12 \sqrt{3} \log (2+\sqrt{3})}. \end{aligned}$$

Precise results also follow for non-badly approximable irrationals whose continued fraction expansions are explicitly known. Consider Euler’s number $e=[2;1,2,1,1,4,1,\dots , 1,2n,1,\dots ]$ as an illustration. Since the “period length” is odd, the square of the alternating sum $(\sum _{k=1}^K (-1)^k a_k)^2 \ll K^2$ is negligible compared to $\sum _{k=1}^K a_k^2 = (4/81)K^3+O(K^2)$. Thus from our general results it easily follows that

$$\begin{aligned} D_2 (S(e,N)) = \frac{1}{3\sqrt{30}} \left( \frac{\log N}{\log \log N} \right) ^{3/2} \left( 1 + O \left( \frac{\log \log \log N}{\log \log N} \right) \right) , \end{aligned}$$

and

$$\begin{aligned} D_2 (L(e,N)) = \frac{1}{6\sqrt{5}} \left( \frac{\log N}{\log \log N} \right) ^{3/2} \left( 1 + O \left( \frac{\log \log \log N}{\log \log N} \right) \right) . \end{aligned}$$

In contrast, e.g. for $\tan 1 = [1;1,1,3,1,5,1,\dots , 2n-1,1, \dots ]$, the “period length” is even, and the alternating sum $(\sum _{k=1}^K (-1)^k a_k)^2=K^4/16+O(K^3)$ dominates $\sum _{k=1}^K a_k^2 = K^3/6 + O(K^2)$. Consequently,

$$\begin{aligned} D_2 (S(\tan 1,N)) = \frac{1}{3\sqrt{30}} \left( \frac{\log N}{\log \log N} \right) ^{3/2} \left( 1 + O \left( \frac{\log \log \log N}{\log \log N} \right) \right) , \end{aligned}$$

but for the unsymmetrized lattice we have the larger order of magnitude

$$\begin{aligned} D_2 (L(\tan 1, N)) = \frac{1}{12} \left( \frac{\log N}{\log \log N} \right) ^2 \left( 1 + O \left( \frac{\log \log \log N}{\log \log N} \right) \right) . \end{aligned}$$

We also establish precise results for randomly chosen $\alpha$, starting with the asymptotics a.e. in the sense of the Lebesgue measure.

### Theorem 3

Let $\varphi$ be a positive nondecreasing function on $(0,\infty )$.

(i) If $\sum _{n=1}^{\infty } 1/\varphi (n) < \infty$, then for a.e. $\alpha$,
  $$\begin{aligned} \begin{aligned} D_2(S(\alpha , N))&\le \varphi (\log N) + O(\log N \log \log N), \\ D_2(L(\alpha , N))&\le \varphi (\log N) + O(\log N \log \log N) \end{aligned} \end{aligned}$$
  with implied constants depending only on $\alpha$ and $\varphi$.
(ii) If $\sum _{n=1}^{\infty } 1/\varphi (n) = \infty$, then for a.e. $\alpha$,
  $$\begin{aligned} D_2 (S(\alpha , N)) \ge \varphi (\log N) \quad \text {and} \quad D_2 (L(\alpha , N)) \ge \varphi (\log N) \quad \text {for infinitely many } N. \end{aligned}$$

In particular, for a.e. $\alpha$ we have $D_2 (S(\alpha , N)) \ll \log N (\log \log N)^{1+\varepsilon }$ and $D_2 (L(\alpha , N)) \ll \log N (\log \log N)^{1+\varepsilon }$ with any $\varepsilon >0$, but these fail with $\varepsilon =0$.

Our next result is the distributional analogue of Theorem 3, stating that if $\alpha$ is chosen randomly from [0, 1] with an absolutely continuous distribution, then after suitable normalization $D_2^2(S(\alpha , N))$ converges to the standard Lévy distribution. If $\alpha$ is chosen randomly with the Lebesgue measure $\lambda$ or the Gauss measure $\nu (B)=(1/\log 2) \int _B 1/(1+x) \, \textrm{d}x$ ($B \subseteq [0,1]$ Borel) as distribution, then we also estimate the rate of convergence in the Kolmogorov metric.

### Theorem 4

If $\mu$ is a Borel probability measure on [0, 1] which is absolutely continuous with respect to the Lebesgue measure, then for any $t \ge 0$,

$$\begin{aligned} \mu \left( \left\{ \alpha \in [0,1] \,: \, 5 \pi ^3 \frac{D_2^2 (S(\alpha , N))}{(\log N)^2} \le t \right\} \right) \rightarrow \int _0^t \frac{e^{-1/(2x)}}{\sqrt{2 \pi } x^{3/2}} \, \textrm{d} x \qquad \text {as } N \rightarrow \infty . \end{aligned}$$

If $\mu$ is either the Lebesgue measure $\lambda$ or the Gauss measure $\nu$, then for any $N \ge 3$,

$$\begin{aligned} \sup _{t \ge 0} \left| \mu \left( \left\{ \alpha \in [0,1] \,: \, 5 \pi ^3 \frac{D_2^2 (S(\alpha , N))}{(\log N)^2} \le t \right\} \right) - \int _0^t \frac{e^{-1/(2x)}}{\sqrt{2 \pi } x^{3/2}} \, \textrm{d} x \right| \ll \frac{(\log \log N)^{1/3}}{(\log N)^{1/3}} \end{aligned}$$

with a universal implied constant.

We conjecture that a similar result holds for the unsymmetrized lattice as well, i.e. if $\alpha$ is chosen randomly from [0, 1] with an absolutely continuous distribution, then $D_2^2(L(\alpha , N))/(\log N)^2$ has a nondegenerate limit distribution as $N \rightarrow \infty$.

Our results, especially Theorems 1, 3 and 4 should be compared to the corresponding properties of the extreme discrepancy $D_{\textrm{extr},N} (n \alpha ) :=D_{\textrm{extr}} (\{ \{n \alpha \} \,: \, 1 \le n \le N \})$ of the classical Kronecker sequence $\{ n \alpha \}$. Note that $\max _{1 \le \ell \le N} D_{\textrm{extr},\ell } (n \alpha )$ is, up to a factor of 2, equal to $D_{\textrm{extr}}(L(\alpha , N))$. Roughly speaking, for $N \approx q_K$ we have $\max _{1 \le \ell \le N} D_{\textrm{extr},\ell }( n \alpha ) \approx \sum _{k=1}^K a_k$. We can characterize all irrationals for which the optimal rate $\log N$ is attained as [16, p. 53]

$$\begin{aligned} D_{\textrm{extr},N} (n \alpha ) \ll \log N \,\, \Longleftrightarrow \,\, \frac{1}{K} \sum _{k=1}^K a_k \ll 1. \end{aligned}$$

The extreme discrepancy $D_{\textrm{extr},N}(n \alpha )$ is also known to satisfy the same asymptotics a.e. as in Theorem 3 [16, p. 63]. A fortiori, the previous two results apply also to $\max _{1 \le \ell \le N} D_{\textrm{extr},\ell }(n \alpha )$, and hence to $D_{\textrm{extr}}(L(\alpha , N))$. We mention two distributional analogues due to Kesten [22]:

$$\begin{aligned} \begin{aligned} \frac{D_{\textrm{extr},N}(n \alpha )}{\log N \log \log N}&\rightarrow \frac{2}{\pi ^2} \quad \text {in measure,} \\ \frac{\max _{1 \le \ell \le N}D_{\textrm{extr},\ell } (n \alpha )}{\log N \log \log N}&\rightarrow \frac{3}{\pi ^2} \quad \text {in measure.} \end{aligned} \end{aligned}$$

Full limit laws for $D_{\textrm{extr},N}(n \alpha )$, $\max _{1 \le \ell \le N} D_{\textrm{extr},\ell } (n \alpha )$ and $D_{\textrm{extr}}(L(\alpha ,N))$ remain challenging open problems.

As a curious observation, we mention that there exists an irrational $\alpha$ such that

$$\begin{aligned} \log N \ll D_2(S(\alpha , N)) \le D_{\textrm{extr}} (S(\alpha , N)) \ll \log N, \end{aligned}$$

and

$$\begin{aligned} \log N \ll D_2(L(\alpha , N)) \le D_{\textrm{extr}} (L(\alpha , N)) \ll \log N, \end{aligned}$$

i.e. both $S(\alpha , N)$ and $L(\alpha , N)$ have optimal extreme discrepancy, but neither has optimal $L^2$ discrepancy. Indeed, it is easy to construct a sequence of positive integers $a_k$ such that $K^{-1} \sum _{k=1}^K a_k \ll 1$ but $\sum _{k=1}^K a_k^2 \gg K^2$ (e.g. let $a_k=k$ if *k* is a power of 2, and $a_k=1$ otherwise).

Consider now the case of a rational $\alpha$. For the sake of simplicity, we will always assume that *N* is the denominator of $\alpha$. That is, given a reduced fraction *p*/*q*, we study the *q*-element set

$$\begin{aligned} L(p/q,q) = \left\{ \left( \left\{ \frac{np}{q} \right\} , \frac{n}{q} \right) \in [0,1)^2 \,: \, 0 \le n \le q-1 \right\} , \end{aligned}$$

and the 2*q*-element set

$$\begin{aligned} S(p/q,q) = \left\{ \left( \left\{ \pm \frac{np}{q} \right\} , \frac{n}{q} \right) \in [0,1)^2 \,: \, 0 \le n \le q-1 \right\} . \end{aligned}$$

Note that *L*(*p*/*q*, *q*) is the 2-dimensional Korobov lattice $\mathcal {L}_{p,1,q}$. The characterization of all sets of rationals for which the $L^2$ discrepancy is optimal is exactly the same as in the irrational case.

### Theorem 5

Let $\mathcal {R} \subseteq \mathbb {Q}$ be an arbitrary set of reduced fractions $p/q=[a_0;a_1,\dots , a_r]$. We have

$$\begin{aligned} \begin{aligned} \sup _{p/q \in \mathcal {R}} \frac{D_2(S(p/q,q))}{\sqrt{\log q}}<\infty \,\, \Longleftrightarrow \,\,&\sup _{p/q \in \mathcal {R}} \frac{1}{r} \sum _{k=1}^r a_k^2< \infty , \\ \sup _{p/q \in \mathcal {R}} \frac{D_2(L(p/q,q))}{\sqrt{\log q}}< \infty \,\, \Longleftrightarrow \,\,&\sup _{p/q \in \mathcal {R}} \frac{1}{r} \sum _{k=1}^r a_k^2< \infty \\&\quad \text { and } \sup _{p/q \in \mathcal {R}} \frac{1}{\sqrt{r}} \left| \sum _{k=1}^r (-1)^k a_k \right| < \infty . \end{aligned} \end{aligned}$$

As an analogue of the metric results on typical values of $\alpha$ in the sense of the Lebesgue measure above, we also study the $L^2$ discrepancy for typical values of rationals. In this case, “typical” means choosing *p*/*q* randomly from the set of Farey fractions, that is, the set of all reduced rationals with bounded denominator.

### Theorem 6

Let $\mathcal {F}_Q$ denote the set of all reduced fractions in the interval (0, 1) with denominator at most *Q*. For any $Q \ge 2$,

$$\begin{aligned} \sup _{t \ge 0} \left| \frac{1}{|\mathcal {F}_Q|} \left| \left\{ \frac{p}{q} \in \mathcal {F}_Q \,: \, 5 \pi ^3 \frac{D_2^2 (S(p/q,q))}{(\log q)^2} \le t \right\} \right| - \int _0^t \frac{e^{-1/(2x)}}{\sqrt{2 \pi } x^{3/2}} \, \textrm{d} x \right| \ll \frac{1}{(\log Q)^{1/2}} \end{aligned}$$

with a universal implied constant.

We conjecture that a similar result holds for the unsymmetrized lattice as well, i.e. if *p*/*q* is chosen randomly from $\mathcal {F}_Q$, then $D_2^2(L(p/q,q))/(\log q)^2$ has a nondegenerate limit distribution as $Q \rightarrow \infty$.

In Sect. 2, we derive an explicit formula for $D_2(S(\alpha , N))$ and $D_2(L(\alpha , N))$ in terms of the partial quotients of $\alpha$, see Propositions 7 and 8. Theorems 1, 2 and 5 are proved in Sect. 2.2. In Sect. 3, we show how Theorems 3 and 4 follow from classical results on the metric theory of continued fractions and $\psi$-mixing random variables. The proof of Theorem 6 in Sect. 4, on the other hand, relies on recent results of Bettin and Drappeau [4] on the statistics of partial quotients of random rationals.

## $L^2$ discrepancy via the Parseval formula

### The main estimates

We remind that $\alpha = [a_0;a_1,a_2, \dots ]$ is the (finite or infinite) continued fraction expansion of a real number $\alpha$, and $p_k/q_k=[a_0;a_1,\dots , a_k]$ denotes its convergents. For the rest of the paper, we also use the notation

$$\begin{aligned} T_n=\sum _{\ell =0}^n \left( \frac{1}{2} - \{ \ell \alpha \} \right) \quad \text {and} \quad E_N=\frac{1}{N}\sum _{n=0}^{N-1} T_n. \end{aligned}$$

Finally, $\zeta$ is the Riemann zeta function.

Our main tool is an evaluation of the $L^2$ discrepancy up to a small error, based on the Parseval formula. This method goes back to Davenport [12], and more recently has also been used in [5–7, 18, 25]. We follow the steps in our previous paper [9], where we considered irrationals whose sequence of partial quotients is reasonably well-behaved (e.g. bounded, or increasing at a regular rate such as for Euler’s number). Here we shall need a more refined analysis in order to study arbitrary reals without any assumption on the partial quotients.

#### Proposition 7

For any $q_{K-1} \le N \le q_K$, we have

$$\begin{aligned}{} & {} \left| D_2^2 (S(\alpha , N)) - \sum _{m=1}^{q_{K-1}-1} \frac{1}{4 \pi ^4 m^2 \Vert m \alpha \Vert ^2} - \xi _S(\alpha , N) \right| \\ {}{} & {} \quad \le \sum _{k=0}^{K-1} \frac{a_{k+1}}{2q_k} + \frac{\zeta (3)}{16 \pi ^4 N} \sum _{k=0}^{K-2} (a_{k+1}+2)^3 q_k + 6.28 \end{aligned}$$

with some $\xi _S(\alpha , N)$ which satisfies both $0 \le \xi _S(\alpha , N) \le \sum _{m=q_{K-1}}^{q_K-1} \frac{1}{2 \pi ^4\,m^2 \Vert m \alpha \Vert ^2}$ and

$$\begin{aligned} \left| \xi _S(\alpha , N) - \sum _{m=q_{K-1}}^{q_K-1} \frac{1}{4 \pi ^4 m^2 \Vert m \alpha \Vert ^2} \right| \le \frac{\zeta (3)}{16 \pi ^4 N} (a_K+2)^3 q_{K-1} +0.07. \end{aligned}$$

Similarly, for any $q_{K-1} \le N \le q_K$, we have

$$\begin{aligned}&\left| D_2^2(L(\alpha , N)) - \frac{1}{N} \sum _{n=0}^{N-1} \left( T_n^2 + \frac{1}{2}T_n \right) - \left( 1-\frac{1}{2N} \right) \sum _{m=1}^{q_{K-1}-1} \frac{1}{4 \pi ^4 m^2 \Vert m \alpha \Vert ^2} - \xi _L(\alpha , N) \right| \\ {}&\qquad \quad \le \sum _{k=0}^{K-1} \frac{a_{k+1}}{8 q_k} + \frac{\zeta (3)}{16 \pi ^4 N} \sum _{k=0}^{K-2} (a_{k+1}+2)^3 q_k + 2.78 \end{aligned}$$

with some $\xi _L(\alpha , N)$ which satisfies both $0 \le \xi _L(\alpha , N) \le \sum _{m=q_{K-1}}^{q_K-1} \frac{1}{2 \pi ^4\,m^2 \Vert m \alpha \Vert ^2}$ and

$$\begin{aligned} \left| \xi _L(\alpha , N) - \left( 1-\frac{1}{2N} \right) \sum _{m=q_{K-1}}^{q_K-1} \frac{1}{4 \pi ^4 m^2 \Vert m \alpha \Vert ^2} \right| \le \frac{\zeta (3)}{16 \pi ^4 N} (a_K+2)^3 q_{K-1}. \end{aligned}$$

We also prove a simpler form which is sharp up to a constant factor.

#### Proposition 8

For any $q_{K-1} \le N \le q_K$, we have $D_2^2 (S(\alpha , N)) \ll \sum _{k=1}^K a_k^2$. For $N=q_K$, we also have $D_2^2 (S(\alpha , q_K)) \gg \sum _{k=1}^K a_k^2$, and

$$\begin{aligned} \sum _{k=1}^K a_k^2 + \left( \sum _{k=1}^K (-1)^k a_k \right) ^2 \ll D_2^2 (L(\alpha , q_K)) \ll \sum _{k=1}^K a_k^2 + \left( \sum _{k=1}^K (-1)^k a_k \right) ^2. \end{aligned}$$

The implied constants are universal.

We postpone the proofs to Sects. 2.3 and 2.4, and now comment on the main terms.

The contribution of the sums $T_n$ can be written as

$$\begin{aligned} \frac{1}{N} \sum _{n=0}^{N-1} \left( T_n^2 + \frac{1}{2} T_n \right) = \frac{1}{N} \sum _{n=0}^{N-1} (T_n-E_N)^2 + E_N^2 + \frac{1}{2} E_N. \end{aligned}$$

Observing a connection with Dedekind sums, Beck showed [1, p. 79 and p. 91] (see also [28]) that for any $q_{K-1} \le N \le q_K$, the “expected value” $E_N$ is

1 $$\begin{aligned} E_N = \frac{1}{12} \sum _{k=1}^K (-1)^k a_k +O \left( \max _{1 \le k \le K} a_k \right) . \end{aligned}$$

For $N=q_K$, the error term can be improved to

2 $$\begin{aligned} E_{q_K} = \frac{1}{12} \sum _{k=1}^K (-1)^k a_k +O(1) . \end{aligned}$$

Both implied constants are universal. Generalizing results of Beck, in a recent paper [8] we proved that if $a_k \le c k^d$ with some constants $c>0$ and $d \ge 0$, then for any $q_{K-1} \le N \le q_K$, the “variance” is

3 $$\begin{aligned} \frac{1}{N} \sum _{n=0}^{N-1} (T_n-E_N)^2 = \sum _{m=1}^{q_K-1} \frac{1}{8 \pi ^4 m^2 \Vert m \alpha \Vert ^2} +O \left( \max _{|k-K| \ll \log K} a_k^2 \cdot (\log \log N)^4 \right) \end{aligned}$$

with implied constants depending only on *c* and *d*. See also Lemma 10 below.

Finally, we will need two different evaluations of the Diophantine sum appearing in Proposition 7. On the one hand, for general $\alpha$ we have [10, p. 110], [9]

4 $$\begin{aligned} \left| \sum _{m=1}^{q_K-1} \frac{1}{m^2 \Vert m \alpha \Vert ^2} - \frac{\pi ^4}{90} \sum _{k=1}^K a_k^2 \right| \le 152 \sum _{k=1}^K a_k . \end{aligned}$$

On the other hand, Beck [1, p. 176] proved that if $\alpha$ is quadratic irrational, then for any $M \ge 1$,

5 $$\begin{aligned} \sum _{m=1}^M \frac{1}{4 \pi ^4 m^2 \Vert m \alpha \Vert ^2} = c(\alpha ) \log M +O(1) \end{aligned}$$

with some constant $c(\alpha )>0$ and an implied constant depending only on $\alpha$.

### Optimal lattices

In this section, we deduce Theorems 1, 2 and 5 from Propositions 7 and 8.

#### Proof of Theorem 1

Consider first the symmetrized lattice $S(\alpha , N)$. We will show the implications

$$\begin{aligned} \frac{1}{K} \sum _{k=1}^K a_k^2 \ll 1 \,\,{} & {} \Longrightarrow \,\, D_2(S(\alpha , N)) \ll \sqrt{\log N} \,\,\\ {}{} & {} \Longrightarrow \,\, D_2(S(\alpha , q_K)) \ll \sqrt{\log q_K} \,\, \Longrightarrow \,\, \frac{1}{K} \sum _{k=1}^K a_k^2 \ll 1. \end{aligned}$$

Assume that $K^{-1} \sum _{k=1}^K a_k^2 \ll 1$ as $K \rightarrow \infty$. By Proposition 8, for any $q_{K-1} \le N \le q_K$ we have $D_2^2 (S(\alpha , N)) \ll \sum _{k=1}^K a_k^2 \ll K \ll \log N$, as claimed. The second implication is trivial. Next, assume that $D_2 (S(\alpha , q_K)) \ll \sqrt{\log q_K}$ as $K \rightarrow \infty$. By Proposition 8, we have

$$\begin{aligned} \sum _{k=1}^K a_k^2 \ll D_2^2 (S(\alpha , q_K)) \ll \log q_K \le \sum _{k=1}^K \log (a_k+1) \ll \sum _{k=1}^K a_k \le \sqrt{K \sum _{k=1}^K a_k^2}, \end{aligned}$$

and the claim follows. This finishes the proof of the equivalence for $S(\alpha , N)$.

Consider now the unsymmetrized lattice $L(\alpha , q_K)$. Assume that $K^{-1} \sum _{k=1}^K a_k^2 \ll 1$ and $K^{-1/2} |\sum _{k=1}^K (-1)^k a_k | \ll 1$ as $K \rightarrow \infty$. By Proposition 8, we have

$$\begin{aligned} D_2^2 (L(\alpha , q_K)) \ll \sum _{k=1}^K a_k^2 + \left( \sum _{k=1}^K (-1)^k a_k \right) ^2 \ll K \ll \log q_K, \end{aligned}$$

as claimed. Next, assume that $D_2(L(\alpha , q_K)) \ll \sqrt{\log q_K}$ as $K \rightarrow \infty$. By Proposition 8, we have

$$\begin{aligned} \sum _{k=1}^K a_k^2 + \left( \sum _{k=1}^K (-1)^k a_k \right) ^2\ll & {} D_2^2(L(\alpha , q_K)) \ll \log q_K \\\le & {} \sum _{k=1}^K \log (a_k+1) \ll \sum _{k=1}^K a_k \le \sqrt{K \sum _{k=1}^K a_k^2}. \end{aligned}$$

In particular,

$$\begin{aligned} \sum _{k=1}^K a_k^2 \ll \sqrt{K \sum _{k=1}^K a_k^2} \qquad \text {and} \qquad \left( \sum _{k=1}^K (-1)^k a_k \right) ^2 \ll \sqrt{K \sum _{k=1}^K a_k^2}. \end{aligned}$$

The first estimate gives $\sum _{k=1}^K a_k^2 \ll K$, whereas the second estimate yields $(\sum _{k=1}^K (-1)^k a_k)^2 \ll K$, as claimed. This finishes the proof of the equivalence for $L(\alpha , q_K)$. $\square$

#### Proof of Theorem 5

As Proposition 8 applies to both rationals and irrationals, the proof is identical to that of Theorem 1. $\square$

#### Proof of Theorem 2

Let $\alpha$ be a quadratic irrational. By Proposition 7 and formula (5), for any $q_{K-1} \le N \le q_K$,

$$\begin{aligned} D_2^2 (S(\alpha , N)) = \sum _{m=1}^{q_K-1} \frac{1}{4 \pi ^4 m^2 \Vert m \alpha \Vert ^2} +O(1)=c(\alpha ) \log N +O(1), \end{aligned}$$

as claimed. Using also formula (3), we similarly get

$$\begin{aligned} D_2^2 (L(\alpha , N)) = \frac{3}{2} c(\alpha ) \log N + E_N^2+\frac{1}{2} E_N +O((\log \log N)^4). \end{aligned}$$

Formula (1) shows that here $E_N= \frac{A(\alpha )}{12} K+O(1)=\frac{A(\alpha )}{12 \Lambda (\alpha )} \log N +O(1)$, and the claim follows. $\square$

### Proof of Proposition 7

#### Lemma 9

Let $\alpha = [a_0;a_1,a_2,\ldots ]$ be the (finite or infinite) continued fraction expansion of a real number $\alpha$, and let $p_k/q_k=[a_0;a_1,\ldots , a_k]$ be its convergents.

(i) For any $$K \ge 1$$,
  $$\begin{aligned} \sum _{m=1}^{q_K-1} \frac{1}{\pi ^2 m^2 \Vert m \alpha \Vert } \le \sum _{k=0}^{K-1} \frac{a_{k+1}}{2 q_k} + 3.12. \end{aligned}$$
(ii) For any $K \ge 1$ and $n \ge 0$,
  $$\begin{aligned} \sum _{m=q_K}^{\infty } \frac{1}{2 \pi ^2 m^2} \min \left\{ \frac{1}{4 \Vert m \alpha \Vert ^2}, n^2 \right\} \le 1.12 \frac{n}{q_K} + 0.61 \frac{n^2}{q_K^2}. \end{aligned}$$
(iii) For any $K \ge 1$ and $N \ge q_{K-1}$,
  $$\begin{aligned} \sum _{m=1}^{q_K-1} \frac{1}{4 \pi ^4 m^2 \Vert m \alpha \Vert ^2} \min \left\{ \frac{1}{4 N \Vert 2 m \alpha \Vert }, 1 \right\} \le \frac{\zeta (3)}{16 \pi ^4 N} \sum _{k=0}^{K-1} (a_{k+1}+2)^3 q_k+ 0.07. \end{aligned}$$

#### Proof

The proof of all three claims is based on the following simple observations. Let $k \ge 1$, or $k=0$ and $a_1>1$. For any integer $a \ge 1$ let $J_{k,a}=[aq_k, (a+1)q_k) \cap [q_k,q_{k+1})$ be a (possibly empty) index set. Let $\delta _k=q_k \alpha -p_k$, and recall from the general theory of continued fractions that $1/(q_{k+1}+q_k) \le |\delta _k|=\Vert q_k \alpha \Vert \le 1/q_{k+1}$. For any integer $m \in J_{k,a}$, we have $m \alpha = mp_k/q_k + m \delta _k/q_k$, and here the second term is negligible as $m|\delta _k|/q_k<1/q_k$. Since $p_k$ and $q_k$ are relatively prime, as *m* runs in the index set $J_{k,a}$, the numbers $mp_k$ attain each mod $q_k$ residue class at most once. If $mp_k \not \equiv 0, \pm 1 \pmod {q_k}$, then

$$\begin{aligned} \Vert m \alpha \Vert = \left\| \frac{mp_k}{q_k} + \frac{m \delta _k}{q_k} \right\| \ge \left\| \frac{mp_k}{q_k} \right\| - \frac{1}{q_k} \ge \frac{1}{2} \left\| \frac{mp_k}{q_k} \right\| . \end{aligned}$$

Therefore for any nondecreasing function $f: [2,\infty ) \rightarrow [0,\infty )$, we have

6 $$\begin{aligned} \sum _{m \in J_{k,a}} f \left( \frac{1}{\Vert m \alpha \Vert } \right)&\le 3 f \left( \frac{1}{\Vert q_k \alpha \Vert } \right) + \sum _{j=2}^{q_k-2} f \left( \frac{2}{\Vert j/q_k \Vert } \right) \nonumber \\&\le 3f \left( \frac{1}{\Vert q_k \alpha \Vert } \right) + 2 \sum _{2 \le j \le q_k/2} f \left( \frac{2q_k}{j} \right) . \end{aligned}$$

Note that $3 f(1/\Vert q_k \alpha \Vert )$ is an upper bound to the contribution of the three terms for which $m p_k \equiv 0, \pm 1 \pmod {q_k}$.

We also have the simpler estimate

7 $$\begin{aligned} \sum _{1 \le m < q_{k+1}} f \left( \frac{1}{\Vert m \alpha \Vert } \right) \le 2 \sum _{1 \le j \le q_{k+1}/2} f \left( \frac{1}{j \Vert q_k \alpha \Vert } \right) . \end{aligned}$$

Indeed, consider the points $m \alpha \pmod {1}$, $1 \le m < q_{k+1}$ and the intervals $H_j=[j \Vert q_k \alpha \Vert , (j+1)\Vert q_k \alpha \Vert )$, $j \ge 1$ and $H_j = ((j-1) \Vert q \alpha \Vert , j \Vert q_k \alpha \Vert ]$, $j \le -1$. Since $\Vert (m_1-m_2) \alpha \Vert \ge \Vert q_k \alpha \Vert$ for any $m_1, m_2 \in [1,q_{k+1})$, $m_1 \ne m_2$, each interval $H_j$ contains at most one point $m \alpha \pmod {1}$, and (7) follows.

(i) Estimate (6) yields
  $$\begin{aligned} \sum _{m \in J_{k,a}} \frac{1}{\pi ^2 m^2 \Vert m \alpha \Vert }\le & {} \frac{1}{\pi ^2 a^2 q_k^2} \left( \frac{3}{\Vert q_k \alpha \Vert } + 2 \sum _{2 \le j \le q_k/2} \frac{2q_k}{j} \right) \\\le & {} \frac{1}{\pi ^2 a^2 q_k^2} \left( 3(q_{k+1}+q_k) + 4 q_k \log \frac{q_k}{2} \right) . \end{aligned}$$
  Summing over $a \ge 1$ and^2^$0 \le k \le K-1$ leads to
  $$\begin{aligned} \begin{aligned} \sum _{m=1}^{q_K-1} \frac{1}{\pi ^2 m^2 \Vert m \alpha \Vert }&\le \sum _{k=0}^{K-1} \frac{3 q_{k+1} + 3q_k + 4 q_k \log (q_k/2)}{6 q_k^2} \\ {}&\le \sum _{k=0}^{K-1} \frac{a_{k+1}}{2 q_k} + \sum _{k=0}^{K-1} \frac{3+2\log (q_k/2)}{3 q_k} \\ {}&\le \sum _{k=0}^{K-1} \frac{a_{k+1}}{2 q_k} + \sum _{k=0}^{\infty } \frac{3+2 \log (F_{k+1}/2)}{3 F_{k+1}}, \end{aligned} \end{aligned}$$
  where $F_k$ is the sequence of Fibonacci numbers. The numerical value of the series in the previous line is $3.1195\dots$, as claimed.
(ii) Estimate (6) yields
  $$\begin{aligned} \sum _{m \in J_{k,a}} \frac{1}{2 \pi ^2 m^2} \min \left\{ \frac{1}{4 \Vert m \alpha \Vert ^2}, n^2 \right\}{} & {} \le \frac{1}{2 \pi ^2 a^2 q_k^2} \left( 3n^2 + 2 \sum _{j=2}^{\infty } \min \left\{ \frac{q_k^2}{j^2},n^2 \right\} \right) \\{} & {} \le \frac{1}{2 \pi ^2 a^2 q_k^2} \left( 3n^2 + 4 n q_k \right) . \end{aligned}$$
  Note that the contribution of the terms $2 \le j \le \lfloor q_k/n \rfloor +1$ and $j \ge \lfloor q_k/n \rfloor +2$ is at most $n q_k$ each. Summing over $a \ge 1$ and $k \ge K$ leads to
  $$\begin{aligned} \sum _{m=q_K}^{\infty } \frac{1}{2 \pi ^2 m^2} \min \left\{ \frac{1}{4 \Vert m \alpha \Vert ^2}, n^2 \right\} \le \sum _{k=K}^{\infty } \frac{3n^2 + 4nq_k}{12 q_k^2}. \end{aligned}$$
  From the recursion satisfied by $q_k$ one readily sees that $q_{K+\ell } \ge F_{\ell +1} q_K$ for all $\ell \ge 0$, hence the right hand side of the previous formula is at most $c_1 n/q_K + c_2n^2/q_K^2$ with $c_1=\sum _{\ell =0}^{\infty } 1/(3F_{\ell +1}) = 1.1199\dots$ and $c_2=\sum _{\ell =0}^{\infty } 1/(4F_{\ell +1}^2)=0.6065\dots$, as claimed.
(iii) The contribution of all *m* such that $\Vert m \alpha \Vert > 1/4$ is negligible:
  $$\begin{aligned} \sum _{\begin{array}{c} 1 \le m \le q_K-1 \\ \Vert m \alpha \Vert > 1/4 \end{array}} \frac{1}{4 \pi ^4 m^2 \Vert m \alpha \Vert ^2} \min \left\{ \frac{1}{4 N \Vert 2 m \alpha \Vert }, 1 \right\} < \sum _{m=1}^{\infty } \frac{4}{\pi ^4 m^2} = \frac{2}{3 \pi ^2}. \end{aligned}$$
  On the other hand, $\Vert m \alpha \Vert \le 1/4$ implies $\Vert 2 m \alpha \Vert = 2 \Vert m \alpha \Vert$, hence the contribution of all such terms is
  $$\begin{aligned} \sum _{\begin{array}{c} 1 \le m \le q_K-1 \\ \Vert m \alpha \Vert \le 1/4 \end{array}} \frac{1}{4 \pi ^4 m^2 \Vert m \alpha \Vert ^2} \min \left\{ \frac{1}{4 N \Vert 2 m \alpha \Vert }, 1 \right\} \le \sum _{m=1}^{q_K-1} \frac{1}{32 \pi ^4 N m^2 \Vert m \alpha \Vert ^3}. \end{aligned}$$
  Estimate (7) gives
  $$\begin{aligned} \sum _{q_k \le m < q_{k+1}} \frac{1}{32 \pi ^4 N m^2 \Vert m \alpha \Vert ^3} \le \frac{1}{16 \pi ^4 N q_k^2} \sum _{j=1}^{\infty } \frac{1}{j^3 \Vert q_k \alpha \Vert ^3} \le \frac{\zeta (3) (a_{k+1}+2)^3 q_k}{16 \pi ^4 N}. \end{aligned}$$

Summing over $0 \le k \le K-1$, we thus obtain

$$\begin{aligned} \sum _{m=1}^{q_K-1} \frac{1}{4 \pi ^4 m^2 \Vert m \alpha \Vert ^2} \min \left\{ \frac{1}{4 N \Vert 2 m \alpha \Vert }, 1 \right\} \le \sum _{k=0}^{K-1} \frac{\zeta (3) (a_{k+1}+2)^3 q_k}{16 \pi ^4 N} + \frac{2}{3 \pi ^2}. \end{aligned}$$

Here $2/(3 \pi ^2)=0.06754\dots$, as claimed. $\square$

#### Proof of Proposition 7

We give a detailed proof for the symmetrized lattice $S(\alpha , N)$, and then indicate at the end how to modify the proof for the unsymmetrized lattice $L(\alpha , N)$.

Let $B(x,y)=|S(\alpha , N) \cap ([0,x) \times [0,y))|$ denote the number of points of $S(\alpha , N)$ which fall into the box $[0,x) \times [0,y)$. Integrating on the strips $[0,1) \times [n/N,(n+1)/N)$ separately leads to

$$\begin{aligned} D_2^2 (S(\alpha , N)) = \sum _{n=0}^{N-1} \int _0^1 \int _{\frac{n}{N}}^{\frac{n+1}{N}} \left( B(x,y) - 2N xy \right) ^2 \, \textrm{d}y \, \textrm{d}x = M+R+\frac{4}{9} \end{aligned}$$

with

$$\begin{aligned} \begin{aligned} M&:=\frac{1}{N} \sum _{n=0}^{N-1} \int _0^1 \left( B \left( x, \frac{n+1}{N} \right) - 2(n+1)x \right) ^2 \, \textrm{d}x, \\ R&:=\frac{2}{N} \sum _{n=0}^{N-1} \int _0^1 \left( B \left( x, \frac{n+1}{N} \right) - 2(n+1)x \right) x \, \textrm{d}x. \end{aligned} \end{aligned}$$

The function

$$\begin{aligned} B \left( x, \frac{n+1}{N} \right) -2(n+1)x = \sum _{\ell =0}^{n} \left( I_{[0,x)}(\{ \ell \alpha \}) + I_{[0,x)}(\{ -\ell \alpha \}) -2x \right) , \end{aligned}$$

where $$I_{[0,x)}$$ denotes the indicator function of the interval [0, *x*), is mean zero, and has Fourier coefficients

$$\begin{aligned} \begin{aligned} \int _0^1 \left( B \left( x, \frac{n+1}{N} \right) -2(n+1)x \right) e^{-2 \pi i m x} \, \textrm{d}x&= \sum _{\ell =0}^n \frac{\cos (2 \ell m \pi \alpha )}{\pi i m} \\ {}&= \frac{1}{2 \pi i m} \left( \frac{\sin ((2n+1)m \pi \alpha )}{\sin (m \pi \alpha )} + 1 \right) . \end{aligned} \end{aligned}$$

The Fourier coefficients of *x* are $\int _0^1 x e^{-2\pi i m x} \, \textrm{d}x=-1/(2 \pi i m)$, thus by the Parseval formula we have

$$\begin{aligned} R= & {} \frac{2}{N} \sum _{n=0}^{N-1} 2 \sum _{m=1}^{\infty } \frac{1}{2 \pi i m} \left( \frac{\sin ((2n+1)m \pi \alpha )}{\sin (m \pi \alpha )} + 1 \right) \cdot \frac{-1}{2 \pi i m} \\ {}= & {} \frac{1}{N} \sum _{n=0}^{N-1} \sum _{m=1}^{\infty } \frac{\sin ((2n+1) m \pi \alpha )}{\pi ^2 m^2 \sin (m \pi \alpha )} +\frac{1}{6}. \end{aligned}$$

The Parseval formula similarly gives

$$\begin{aligned} \begin{aligned} M&= \frac{1}{N} \sum _{n=0}^{N-1} 2 \sum _{m=1}^{\infty } \frac{1}{4 \pi ^2 m^2} \left( \frac{(\sin ((2n+1)m \pi \alpha )}{\sin (m \pi \alpha )} + 1 \right) ^2 \\ {}&= \frac{1}{N} \sum _{n=0}^{N-1} \sum _{m=1}^{\infty } \frac{\sin ^2((2n+1)m \pi \alpha )}{2\pi ^2 m^2 \sin ^2 (m \pi \alpha )} + \frac{1}{N} \sum _{n=0}^{N-1} \sum _{m=1}^{\infty } \frac{\sin ((2n+1)m \pi \alpha )}{\pi ^2 m^2 \sin (m \pi \alpha )} + \frac{1}{12}. \end{aligned} \end{aligned}$$

The only main term in $D_2^2 (S(\alpha , N))$ is the first double sum in the previous formula. The double sum in *R* and the second double sum in the previous formula are identical. Using

$$\begin{aligned} \left| \frac{\sin ((2n+1)m \pi \alpha )}{\sin (m \pi \alpha )} \right| \le \min \left\{ \frac{1}{2 \Vert m \alpha \Vert }, 2n+1 \right\} \end{aligned}$$

and Lemma 9 (i), they can be estimated as

$$\begin{aligned} \begin{aligned} \bigg | \frac{1}{N} \sum _{n=0}^{N-1} \sum _{m=1}^{\infty } \frac{2 \sin ((2n+1)m \pi \alpha )}{\pi ^2 m^2 \sin (m \pi \alpha )} \bigg |&\le \frac{1}{N} \sum _{n=0}^{N-1} \left( \sum _{m=1}^{q_K-1} \frac{1}{\pi ^2 m^2 \Vert m \alpha \Vert } + \sum _{m=q_K}^{\infty } \frac{2(2n+1)}{\pi ^2 m^2} \right) \\ {}&\le \sum _{k=0}^{K-1} \frac{a_{k+1}}{2q_k} +3.12+ \frac{4N}{\pi ^2 q_K}. \end{aligned} \end{aligned}$$

By the assumption $N \le q_K$ and the fact $3.12+4/\pi ^2+4/9+1/6+1/12 <4.22$, we thus obtain

$$\begin{aligned} \left| D_2^2 (S(\alpha , N)) - \frac{1}{N} \sum _{n=0}^{N-1} \sum _{m=1}^{\infty } \frac{\sin ^2((2n+1)m \pi \alpha )}{2\pi ^2 m^2 \sin ^2 (m \pi \alpha )} \right| \le \sum _{k=0}^{K-1} \frac{a_{k+1}}{2q_k} + 4.22. \end{aligned}$$

Lemma 9 (ii) estimates the tail of the infinite series in the previous formula as

$$\begin{aligned} \sum _{m=q_K}^{\infty } \frac{\sin ^2((2n+1)m \pi \alpha )}{2\pi ^2 m^2 \sin ^2 (m \pi \alpha )}{} & {} \le \sum _{m=q_K}^{\infty } \frac{1}{2 \pi ^2 m^2} \min \left\{ \frac{1}{4 \Vert m \alpha \Vert ^2}, (2n+1)^2 \right\} \\ {}{} & {} \le 1.12 \frac{2n+1}{q_K} + 0.61 \frac{(2n+1)^2}{q_K^2}. \end{aligned}$$

By the assumption $N \le q_K$ and the facts $\sum _{n=0}^{N-1}(2n+1)^2 \le (4/3)N^3$ and $4.22+1.12+(4/3)\cdot 0.61<6.16$, we immediately get

$$\begin{aligned} \left| D_2^2 (S(\alpha , N)) - \frac{1}{N} \sum _{n=0}^{N-1} \sum _{m=1}^{q_K-1} \frac{\sin ^2((2n+1)m \pi \alpha )}{2\pi ^2 m^2 \sin ^2 (m \pi \alpha )} \right| \le \sum _{k=0}^{K-1} \frac{a_{k+1}}{2q_k} + 6.16. \end{aligned}$$

Elementary calculations show that the function $1/\sin ^2 (\pi x) - 1/(\pi ^2 \Vert x \Vert ^2)$ is increasing on (0, 1/2], hence $1/(\pi ^2 \Vert x \Vert ^2) \le 1/\sin ^2 (\pi x) \le 1/(\pi ^2 \Vert x \Vert ^2) + 1-4/\pi ^2$ for all *x*. The error of replacing $\sin ^2 (m \pi \alpha )$ by $\pi ^2 \Vert m \alpha \Vert ^2$ in the denominator of the previous formula is thus at most

$$\begin{aligned} \frac{1}{N} \sum _{n=0}^{N-1} \sum _{m=1}^{q_K-1} \frac{\sin ^2 ((2n+1)m \pi \alpha ) (1-4/\pi ^2)}{2 \pi ^2 m^2} \le \sum _{m=1}^{\infty } \frac{1-4/\pi ^2}{2 \pi ^2 m^2} = \frac{1-4/\pi ^2}{12}. \end{aligned}$$

Since $6.16+(1-4/\pi ^2)/12 <6.21$, we obtain

8 $$\begin{aligned} \left| D_2^2 (S(\alpha , N)) - \frac{1}{N} \sum _{n=0}^{N-1} \sum _{m=1}^{q_{K-1}-1} \frac{\sin ^2((2n+1)m \pi \alpha )}{2\pi ^4 m^2 \Vert m \alpha \Vert ^2} - \xi _S(\alpha , N) \right| \le \sum _{k=0}^{K-1} \frac{a_{k+1}}{2q_k} + 6.21, \end{aligned}$$

where we define

$$\begin{aligned} \xi _S(\alpha , N) :=\frac{1}{N} \sum _{n=0}^{N-1} \sum _{m=q_{K-1}}^{q_K-1} \frac{\sin ^2((2n+1)m \pi \alpha )}{2\pi ^4 m^2 \Vert m \alpha \Vert ^2}. \end{aligned}$$

Using the trigonometric identity

$$\begin{aligned} \frac{1}{N} \sum _{n=0}^{N-1} \sin ^2 ((2n+1)x) = \frac{1}{2} - \frac{\sin (4Nx)}{4N\sin (2x)}, \end{aligned}$$

the double sum in (8) simplifies to

$$\begin{aligned} \sum _{m=1}^{q_{K-1}-1} \frac{1}{4 \pi ^4 m^2 \Vert m \alpha \Vert ^2} - \sum _{m=1}^{q_{K-1}-1} \frac{\sin (4Nm \pi \alpha )}{8 \pi ^4 N m^2 \Vert m \alpha \Vert ^2 \sin (2m\pi \alpha )}. \end{aligned}$$

Here second term can be estimated using Lemma 9 (iii) as

$$\begin{aligned} \begin{aligned} \left| \sum _{m=1}^{q_{K-1}-1} \frac{\sin (4Nm \pi \alpha )}{8 \pi ^4 N m^2 \Vert m \alpha \Vert ^2 \sin (2m\pi \alpha )} \right|&\le \sum _{m=1}^{q_{K-1}-1} \frac{1}{4 \pi ^4 m^2 \Vert m \alpha \Vert ^2} \min \left\{ \frac{1}{4N \Vert 2m \alpha \Vert }, 1 \right\} \\ {}&\le \frac{\zeta (3)}{16 \pi ^4 N} \sum _{k=0}^{K-2} (a_{k+1}+2)^3 q_k + 0.07. \end{aligned} \end{aligned}$$

Therefore (8) simplifies to

$$\begin{aligned} \left| D_2^2 (S(\alpha , N)) - \sum _{m=1}^{q_{K-1}-1} \frac{1}{4 \pi ^4 m^2 \Vert m \alpha \Vert ^2} - \xi _S(\alpha , N) \right|{} & {} \le \sum _{k=0}^{K-1} \frac{a_{k+1}}{2q_k} \\ {}{} & {} \quad + \frac{\zeta (3)}{16 \pi ^4 N} \sum _{k=0}^{K-2} (a_{k+1}+2)^3 q_k + 6.28, \end{aligned}$$

and it remains to prove the properties of $\xi _S(\alpha , N)$. Clearly, $0 \le \xi _S(\alpha , N) \le \sum _{m=q_{K-1}}^{q_K-1} \frac{1}{2 \pi ^4\,m^2 \Vert m \alpha \Vert ^2}$. On the other hand, repeating arguments from above and from Lemma 9 (iii), we can also write

$$\begin{aligned} \xi _S(\alpha , N) = \sum _{m=q_{K-1}}^{q_K-1} \frac{1}{4 \pi ^4 m^2 \Vert m \alpha \Vert ^2} - \sum _{m=q_{K-1}}^{q_K-1} \frac{\sin (4Nm \pi \alpha )}{8 \pi ^4 N m^2 \Vert m \alpha \Vert ^2 \sin (2m\pi \alpha )}, \end{aligned}$$

consequently

$$\begin{aligned} \begin{aligned} \left| \xi _S(\alpha , N) - \sum _{m=q_{K-1}}^{q_K-1} \frac{1}{4 \pi ^4 m^2 \Vert m \alpha \Vert ^2} \right|&\le \sum _{m=q_{K-1}}^{q_K-1} \frac{1}{4 \pi ^4 m^2 \Vert m \alpha \Vert ^2} \min \left\{ \frac{1}{4N \Vert 2m \alpha \Vert }, 1 \right\} \\ {}&\le \frac{\zeta (3)}{16 \pi ^4 N} (a_K+2)^3 q_{K-1} +0.07. \end{aligned} \end{aligned}$$

This finishes the proof for $S(\alpha , N)$.

The proof for $L(\alpha , N)$ is entirely analogous. The only difference is that the number of points $B(x,y):=|L(\alpha , N) \cap ([0,x) \times [0,y))|$ which fall into the box $[0,x) \times [0,y)$ satisfies

$$\begin{aligned} B \left( x, \frac{n+1}{N} \right) - (n+1) x = \sum _{\ell =0}^n \left( I_{[0,x)}(\{ \ell \alpha \}) -x \right) , \end{aligned}$$

which is not a mean zero function. Its integral (0th Fourier coefficient) is

$$\begin{aligned} \int _0^1 \left( B \left( x, \frac{n+1}{N} \right) - (n+1) x \right) \, \textrm{d} x = \sum _{\ell =0}^n \left( \frac{1}{2} - \{ \ell \alpha \} \right) =T_n, \end{aligned}$$

which introduces the extra terms $N^{-1} \sum _{n=0}^{N-1} T_n/2$ resp. $N^{-1} \sum _{n=0}^{N-1} T_n^2$ when the Parseval formula is applied to the analogue of *R* resp. *M* as above. For the convenience of the reader we mention that the analogue of formula (8) is

$$\begin{aligned} \left| D_2^2 (L(\alpha , N)) - \frac{1}{N} \sum _{n=0}^{N-1} \left( T_n^2+\frac{1}{2} T_n \right) - \frac{1}{N} \sum _{n=0}^{N-1} \sum _{m=1}^{q_{K-1}-1} \frac{\sin ^2((n+1)m \pi \alpha )}{2\pi ^4 m^2 \Vert m \alpha \Vert ^2} - \xi _L(\alpha , N) \right| \\ \le \sum _{k=0}^{K-1} \frac{a_{k+1}}{8q_k} + 2.78, \end{aligned}$$

where

$$\begin{aligned} \xi _L (\alpha , N) :=\frac{1}{N} \sum _{n=0}^{N-1} \sum _{m=q_{K-1}}^{q_K-1} \frac{\sin ^2((n+1)m \pi \alpha )}{2\pi ^4 m^2 \Vert m \alpha \Vert ^2}. \end{aligned}$$

$$\square $$

### Proof of Proposition 8

The following lemma is a simpler form of formula (3), but it applies without any assumption on the partial quotients. As modifying the proof of (3) is not entirely straightforward, we include the details.

#### Lemma 10

For any $$K \ge 1$$,

$$\begin{aligned} \frac{1}{q_K} \sum _{n=0}^{q_K-1} (T_n-E_{q_K})^2 \ll \sum _{k=1}^K a_k^2 \end{aligned}$$

with a universal implied constant.

#### Proof

For the sake of readability, set $p=p_K$ and $q=q_K$. For any integer $1 \le \ell \le q-1$, we have $\Vert \ell p/q \Vert \ge 1/q$ and $|\ell \alpha - \ell p/q| \le q |\alpha -p/q| < 1/q$. Thus there is no integer between $\ell p/q$ and $\ell \alpha$, hence

$$\begin{aligned} \left| \{ \ell \alpha \} - \left\{ \frac{\ell p}{q} \right\} \right| \le \left| \ell \alpha - \frac{\ell p}{q} \right| < \frac{1}{q}. \end{aligned}$$

Consequently, for all $0 \le n \le q-1$,

$$\begin{aligned} T_n=\sum _{\ell =0}^n \left( \frac{1}{2} - \{ \ell \alpha \} \right) = \sum _{\ell =0}^n \left( \frac{1}{2} - \frac{1}{2q} - \left\{ \frac{\ell p}{q} \right\} \right) + O(1). \end{aligned}$$

Introducing

$$\begin{aligned} T_n^* :=\sum _{\ell =0}^n \left( \frac{1}{2} - \frac{1}{2q} - \left\{ \frac{\ell p}{q} \right\} \right) \quad \text {and} \quad E_q^* :=\frac{1}{q} \sum _{n=0}^{q-1} T_n^*, \end{aligned}$$

we thus have $T_n -E_q = T_n^* - E_q^*+O(1)$. Therefore $q^{-1} \sum _{n=0}^{q-1} (T_n-E_q)^2 \ll q^{-1} \sum _{n=0}^{q-1} (T_n^*-E_q^*)^2 +1$, and it remains to estimate the latter.

The rest of the proof is based on Fourier analysis on the finite cyclic group $\mathbb {Z}_q$, which we identify by $\{ 0,1,\dots , q-1 \}$. Elementary calculations show that

$$\begin{aligned} \sum _{x=0}^{q-1} \left( \frac{1}{2} - \frac{1}{2q} - \left\{ \frac{x}{q} \right\} \right) e^{-2 \pi i m x/q} = \left\{ \begin{array}{ll} 0 &{} \text {if } m=0, \\ 1/(1-e^{-2 \pi i m/q}) &{} \text {if } 1 \le m \le q-1. \end{array} \right. \end{aligned}$$

Therefore by Fourier inversion on $\mathbb {Z}_q$,

$$\begin{aligned} \frac{1}{2} - \frac{1}{2q} - \left\{ \frac{x}{q} \right\} = \frac{1}{q} \sum _{m=1}^{q-1} \frac{e^{2 \pi i m x/q}}{1-e^{-2 \pi i m /q}}, \qquad x \in \mathbb {Z}. \end{aligned}$$

We can thus write $T_n^*$ as

$$\begin{aligned} T_n^* = \frac{1}{q} \sum _{m=1}^{q-1} \sum _{\ell =0}^n \frac{e^{2 \pi i m \ell p /q}}{1-e^{-2 \pi i m/q}} = \frac{1}{q} \sum _{m=1}^{q-1} \frac{1-e^{2 \pi i m (n+1)p/q}}{(1-e^{- 2 \pi i m/q}) (1-e^{2 \pi i m p/q})}. \end{aligned}$$

Letting $B=q^{-1} \sum _{m=1}^{q-1} 1/(1-e^{-2 \pi i m/q})(1-e^{2 \pi i mp/q})$, we have

$$\begin{aligned} \frac{1}{q} \sum _{n=0}^{q-1} (T_n^*-E_q^*)^2 \le \frac{1}{q} \sum _{n=0}^{q-1} |T_n^*-B|^2 = \frac{1}{q} \sum _{n=0}^{q-1} \frac{1}{q^2} \left| \sum _{m=1}^{q-1} \frac{e^{2 \pi i m (n+1)p/q}}{(1-e^{-2 \pi i m/q})(1-e^{2 \pi i m p/q})} \right| ^2. \end{aligned}$$

Expanding the square shows that here

$$\begin{aligned} \begin{aligned}&\bigg | \sum _{m=1}^{q-1} \frac{e^{2 \pi i m (n+1)p/q}}{(1-e^{-2 \pi i m/q})(1-e^{2 \pi i m p/q})} \bigg |^2 \\ {}&\quad = \sum _{m=1}^{q-1} \frac{1}{|1-e^{-2 \pi i m/q}|^2 |1-e^{2 \pi i m p/q}|^2} \\ {}&\qquad + \sum _{\begin{array}{c} m_1, m_2=1 \\ m_1 \ne m_2 \end{array}}^{q-1} \frac{e^{2 \pi i (m_1-m_2)(n+1)p/q}}{(1-e^{- 2 \pi i m_1 /q})(1-e^{2 \pi i m_1 p/q})(1-e^{2 \pi i m_2 /q})(1-e^{-2 \pi i m_2 p/q})}. \end{aligned} \end{aligned}$$

As $\sum _{n=0}^{q-1} e^{2 \pi i (m_1-m_2)(n+1)p/q} =0$ for all $m_1 \ne m_2$, the contribution of the off-diagonal terms is zero. Formula (4) thus leads to

$$\begin{aligned} \frac{1}{q} \sum _{n=0}^{q-1} (T_n^*-E_q^*)^2 \le \frac{1}{q^2} \sum _{m=1}^{q-1} \frac{1}{|1-e^{-2 \pi i m/q}|^2 |1-e^{2 \pi i mp/q}|^2} \ll \sum _{m=1}^{q-1} \frac{1}{m^2 \Vert mp/q \Vert ^2} \ll \sum _{k=1}^K a_k^2, \end{aligned}$$

as claimed. $$\square $$

#### Proof of Proposition 8

By Proposition 7, for any $q_{K-1} \le N \le q_K$ we have

$$\begin{aligned} D_2^2(S(\alpha , N)) \ll \sum _{m=1}^{q_K-1} \frac{1}{m^2 \Vert m \alpha \Vert ^2} + \sum _{k=0}^{K-1} \frac{a_{k+1}}{q_k} + \sum _{k=0}^{K-2} \frac{a_{k+1}^3 q_k}{N}. \end{aligned}$$

Here $a_{k+1}^3 q_k /N \le a_{k+1}^2$, hence formula (4) yields $D_2^2(S(\alpha , N)) \ll \sum _{k=1}^K a_k^2$, as claimed. Using Lemma 10 and formula (2) we also deduce that for $N=q_K$,

$$\begin{aligned} \frac{1}{q_K} \sum _{n=0}^{q_K-1} \left( T_n^2+\frac{1}{2} T_n \right) = \frac{1}{q_K} \sum _{n=0}^{q_K-1} (T_n-E_{q_K})^2 + E_{q_K}^2 + \frac{1}{2} E_{q_K} \ll \sum _{k=1}^K a_k^2 + \left( \sum _{k=1}^K (-1)^k a_k \right) ^2, \end{aligned}$$

and the upper bound for $D_2^2(L(\alpha , q_K))$ follows.

Next, we prove the lower bounds. Let $c>0$ resp. $C>0$ denote suitably small resp. large universal constants whose values change from line to line. By Proposition 7 and formula (4), for $N=q_K$ we have

$$\begin{aligned} \begin{aligned} D_2^2 (S(\alpha , q_K))&\ge \sum _{m=1}^{q_K-1} \frac{1}{4 \pi ^4 m^2 \Vert m \alpha \Vert ^2} - \frac{\zeta (3)}{16 \pi ^4 q_K} \sum _{k=0}^{K-1} (a_{k+1}+2)^3 q_k - C \sum _{k=1}^K a_k \\ {}&\ge \left( \frac{1}{360} - \frac{\zeta (3)}{16 \pi ^4} \right) \sum _{k=1}^K a_k^2 -C \sum _{k=1}^K a_k. \end{aligned} \end{aligned}$$

The point is that $1/360>\zeta (3)/(16 \pi ^4)$, i.e. the coefficient of $a_k^2$ is positive. The contribution of all *k* such that $a_k \ll 1$ is $\ll K$, and for all other terms $a_k^2$ dominates $a_k$. Therefore $D_2^2 (S(\alpha , q_K)) \ge c \sum _{k=1}^K a_k^2 -CK$. On the other hand, by Roth’s theorem we also have $D_2^2 (S(\alpha , q_K)) \gg \log q_K \gg K$. Taking a suitable weighted average of the previous two inequalities establishes the lower bound $D_2^2(S(\alpha , q_K)) \ge c \sum _{k=1}^K a_k^2$.

From Proposition 7 we similarly deduce

$$\begin{aligned} D_2^2(L(\alpha , q_K)) \ge \frac{1}{q_K} \sum _{n=1}^{q_K-1} \left( T_n-E_{q_K} \right) ^2 + E_{q_K}^2 + c \sum _{k=1}^K a_k^2. \end{aligned}$$

Here $q_K^{-1} \sum _{n=0}^{q_K-1} (T_n-E_{q_K})^2 \ge 0$, and the lower bound for $D_2^2(L(\alpha , q_K))$ follows from formula (2). $\square$

### Proof of Remark 1

Let $\alpha$ be an irrational such that $a_k \ll \sqrt{k}/(\log k)^2$. For any $q_{K-1} \le N \le q_K$ we then have $\max _{|k-K| \ll \log K} a_k^2 \cdot (\log \log N)^4 \ll K$, hence formulas (3) and (4) give

$$\begin{aligned} \frac{1}{N} \sum _{n=0}^{N-1} (T_n-E_N)^2 = \sum _{m=1}^{q_K-1} \frac{1}{8 \pi ^4 m^2 \Vert m \alpha \Vert ^2} +O(K) \ll \sum _{k=1}^K a_k^2. \end{aligned}$$

Using this fact instead of Lemma 10 in the proof of Proposition 8, we deduce that $D_2^2(L(\alpha , N)) \ll \sum _{k=1}^K a_k^2 + (\sum _{k=1}^K (-1)^k a_k)^2$ holds for all $q_{K-1} \le N \le q_K$ (instead of only for $N=q_K$). In particular, the equivalence stated in Remark 1 follows.

## Typical irrationals

### Asymptotics almost everywhere

Let us recall certain basic facts about the statistics of the partial quotients of a typical irrational number. Let $\varphi$ be a positve nondecreasing function on $(0,\infty )$, and let $A_K=\max _{1 \le k \le K} a_k$. It is well known that for a.e. $\alpha$ we have $\log q_k \sim \frac{\pi ^2}{12 \log 2} k$, and that $a_k \le \varphi (k)$ for all but finitely many *k* if and only if $\sum _{n=1}^{\infty } 1/\varphi (n)< \infty$. A classical result of Diamond and Vaaler [13] on trimmed sums states that for a.e. $\alpha$,

9 $$\begin{aligned} \frac{\sum _{k=1}^K a_k - A_K}{K \log K} \rightarrow \frac{1}{\log 2} \qquad \text {as } K \rightarrow \infty . \end{aligned}$$

#### Proof of Theorem 3

For any $N \ge 2$, let $K_N(\alpha )$ be the positive integer for which $q_{K_N(\alpha ) -1} < N \le q_{K_N(\alpha )}$. In particular, for a.e. $\alpha$ we have $K_N(\alpha ) \sim \frac{12 \log 2}{\pi ^2} \log N$, where $\frac{12 \log 2}{\pi ^2}=0.8427\dots$.

**(i)** Assume that $\sum _{n=1}^{\infty } 1/\varphi (n)<\infty$. As observed in the Introduction, by a classical discrepancy estimate for the sequence $\{ n \alpha \}$ [16, p. 52], we have

$$\begin{aligned} \begin{aligned}&D_2 (S(\alpha , N)) \ll D_{\infty } (L(\alpha , N)) \ll \sum _{k=1}^{K_N(\alpha )} a_k, \\ {}&D_2 (L(\alpha , N)) \ll D_{\infty } (L(\alpha , N)) \ll \sum _{k=1}^{K_N(\alpha )} a_k. \end{aligned} \end{aligned}$$

The asymptotic relation (9) of Diamond and Vaaler shows that for a.e. $\alpha$,

$$\begin{aligned} \begin{aligned}&D_2 (S(\alpha , N)) \le C \sum _{k=1}^{K_N(\alpha )} a_k = C A_{K_N(\alpha )} + O(K_N(\alpha ) \log K_N(\alpha )), \\ {}&D_2 (L(\alpha , N)) \le C \sum _{k=1}^{K_N(\alpha )} a_k = C A_{K_N(\alpha )} + O(K_N(\alpha ) \log K_N(\alpha ) ) \end{aligned} \end{aligned}$$

with a universal constant $C>0$. Here $A_{K_N(\alpha )} \le \varphi (K_N(\alpha ))$ and $K_N(\alpha ) \le \log N$ for all but finitely many *N*. Therefore $D_2(S(\alpha , N)) \le C \varphi (\log N)+O(\log N \log \log N)$ and $D_2(L(\alpha , N)) \le C \varphi (\log N)+O(\log N \log \log N)$ with implied constants depending only on $\alpha$ and $\varphi$. The factor *C* can be removed by repeating the argument with $\varphi (x)/C$ instead of $\varphi (x)$.

**(ii)** Assume that $\sum _{n=1}^{\infty } 1/\varphi (n)=\infty$. By Proposition 8, we have

$$\begin{aligned} D_2(S(\alpha , q_K)) \ge c \bigg ( \sum _{k=1}^K a_k^2 \bigg )^{1/2} \ge c A_K \quad \text {and} \quad D_2(L(\alpha , q_K)) \ge c \bigg ( \sum _{k=1}^K a_k^2 \bigg )^{1/2} \ge c A_K \end{aligned}$$

with a universal constant $c>0$. Here $A_K \ge \varphi (K)$ for infinitely many *K*, and $K \ge (\log q_K)/2$ for all but finitely many *K*. Hence $D_2(S(\alpha , q_K)) \ge c \varphi ((\log q_K)/2)$ and $D_2(L(\alpha , q_K)) \ge c \varphi ((\log q_K)/2)$ for infinitely many *K*. Repeating the argument with $\varphi (2x)/c$ instead of $\varphi (x)$, we deduce that $D_2(S(\alpha , q_K)) \ge \varphi (\log q_K)$ and $D_2(L(\alpha , q_K)) \ge \varphi (\log q_K)$ for infinitely many *K*, as claimed. $\square$

### Limit distribution

Let $\lambda$ be the Lebesgue measure, and $\nu (B)= (1/\log 2) \int _B 1/(1+x) \, \textrm{d} x$ ($B \subseteq [0,1]$ Borel) the Gauss measure. If $\alpha$ is chosen randomly from [0, 1] with distribution $\nu$, then its partial quotients are identically distributed random variables with distribution

$$\begin{aligned} \nu \left( \left\{ \alpha \in [0,1] \,: \, a_k = n \right\} \right) = \frac{1}{\log 2} \log \left( 1+\frac{1}{n(n+2)} \right) , \qquad k,n \ge 1. \end{aligned}$$

If $\alpha$ is chosen randomly from [0, 1] with distribution either $\lambda$ or $\nu$, then the sequence $a_k$ is $\psi$-mixing with exponential rate [21, p. 119].

To find the limit distribution of $D_2^2(S(\alpha , N))/(\log N)^2$, we shall need more sophisticated facts about the partial quotients of a typical irrational, which we now gather. Most importantly, a special case of a limit distribution theorem of Samur [27] (see also [8]) states that if $\mu$ is a Borel probability measure on [0, 1] which is absolutely continuous with respect to the Lebesgue measure, then for any $t \ge 0$,

10 $$\begin{aligned} \mu \left( \left\{ \alpha \in [0,1] \, : \, \frac{2 (\log 2)^2}{\pi K^2} \sum _{k=1}^K a_k^2 \le t \right\} \right) \rightarrow \int _0^t \frac{e^{-1/(2x)}}{\sqrt{2 \pi } x^{3/2}} \, \textrm{d}x \qquad \text {as } K \rightarrow \infty . \end{aligned}$$

If $\mu$ is either $\lambda$ or $\nu$, then general results of Heinrich [17] on $\psi$-mixing random variables imply the rate of convergence

11 $$\begin{aligned} \sup _{t \ge 0} \left| \mu \left( \left\{ \alpha \in [0,1] \, : \, \frac{2 (\log 2)^2}{\pi K^2} \sum _{k=1}^K a_k^2 \le t \right\} \right) - \int _0^t \frac{e^{-1/(2x)}}{\sqrt{2 \pi } x^{3/2}} \, \textrm{d}x \right| \ll \frac{1}{K^{1-\varepsilon }} \end{aligned}$$

with an arbitrary $\varepsilon >0$ and an implied constant depending only on $\varepsilon$. The corresponding result for $\sum _{k=1}^K a_k$ in the Gauss measure is also due to Heinrich:

$$\begin{aligned} \sup _{t \in \mathbb {R}} \left| \nu \left( \left\{ \alpha \in [0,1] \,: \, \frac{1}{K} \sum _{k=1}^K a_k - \frac{\log K -\gamma }{\log 2} \le t \right\} \right) - F(t) \right| \ll \frac{(\log K)^2}{K}, \end{aligned}$$

where $\gamma$ is the Euler–Mascheroni constant, and *F*(*t*) is the distribution function of the law with characteristic function

$$\begin{aligned} \int _{\mathbb {R}} e^{itx} \, \textrm{d}F(t) = \exp \left( - \frac{\pi }{2 \log 2} |x| \left( 1+\frac{2i}{\pi } \textrm{sgn}(x) \log |x| \right) \right) . \end{aligned}$$

Note that this is a stable law with stability parameter 1 (and skewness parameter 1). Hence $1-F(t) \ll 1/t$ as $t \rightarrow \infty$, and we immediately obtain

12 $$\begin{aligned} \nu \left( \left\{ \alpha \in [0,1] \, : \, \frac{1}{K} \sum _{k=1}^K a_k \ge t + \frac{\log K}{\log 2} \right\} \right) \ll \frac{1}{t} + \frac{(\log K)^2}{K} \qquad \text {as } t \rightarrow \infty . \end{aligned}$$

The final ingredient is a similar estimate for the convergent denominators: with a large enough universal constant $C>0$,

13 $$\begin{aligned} \nu \left( \left\{ \alpha \in [0,1] \, : \, \left| \log q_K - \frac{\pi ^2}{12 \log 2} K \right| \ge C \sqrt{K \log K} \right\} \right) \ll \frac{1}{\sqrt{K}} . \end{aligned}$$

This follows from the fact that $\log q_K$ satisfies the central limit theorem with rate $O(1/\sqrt{K})$, as shown by Morita [23]. We mention that a better upper bound can be deduced from the large deviation inequality of Takahasi [29], but (13) suffices for our purposes.

#### Proof of Theorem 4

Throughout the proof, $C>0$ is a large universal constant whose value changes from line to line, and $Y_i=Y_i(\alpha , N)$, $i=1,2,\ldots$ are error terms. For any $N \ge 2$, let $K_N(\alpha )$ be the positive integer for which $q_{K_N(\alpha ) -1} < N \le q_{K_N(\alpha )}$.

Proposition 7 and formula (4) show that we can write

$$\begin{aligned} D_2^2 (S(\alpha , N))&= \dfrac{1}{360} \sum \limits _{k=1}^{K_N(\alpha )-1} a_k^2 + Y_1, \quad \end{aligned}$$

where

$$\begin{aligned} |Y_1| \le \frac{1}{180} a_{K_N(\alpha )}^2+ C \sum _{k=1}^{K_N(\alpha )} a_k + \frac{C}{N} \sum _{k=0}^{K_N(\alpha ) -2} a_{k+1}^3 q_k. \end{aligned}$$

Using the general fact $q_{k+2}/q_k \ge 2$, we estimate the last error term as

$$\begin{aligned} \begin{aligned} \frac{1}{N} \sum _{k=0}^{K_N(\alpha )-2} a_{k+1}^3 q_k&\le \frac{1}{N} \sum _{k=1}^{K_N(\alpha )-1} a_k^2 q_k \\ {}&\le \sum _{k=1}^{K_N(\alpha ) - 100 \log K_N(\alpha )} a_k^2 \frac{q_k}{q_{K_N(\alpha )-1}} + \sum _{k=K_N(\alpha ) - 100 \log K_N(\alpha )}^{K_N(\alpha ) -1} a_k^2 \frac{q_k}{q_{K_N(\alpha ) -1}} \\ {}&\le \frac{1}{K_N(\alpha )^{10}} \sum _{k=1}^{K_N(\alpha )} a_k^2 + \sum _{k=K_N(\alpha ) - 100 \log K_N(\alpha )}^{K_N(\alpha ) -1} a_k^2. \end{aligned} \end{aligned}$$

This leads to the simplified form $D_2^2(S(\alpha , N)) = (1/360) \sum _{k=1}^{K_N(\alpha )} a_k^2 + Y_2$, where

$$\begin{aligned} |Y_2| \le \frac{C}{K_N(\alpha )^{10}} \sum _{k=1}^{K_N(\alpha )} a_k^2 + C\sum _{k=K_N(\alpha ) -100 \log K_N(\alpha )}^{K_N(\alpha )} a_k^2 + C\sum _{k=1}^{K_N(\alpha )} a_k. \end{aligned}$$

Set $\overline{K}=\lceil \frac{12 \log 2}{\pi ^2} \log N \rceil$. The estimate (13) states that

$$\begin{aligned} \nu \left( \left\{ \alpha \in [0,1] \,: \, \left| \log q_{\overline{K}} - \frac{\pi ^2}{12 \log 2} \overline{K} \right| \ge C \sqrt{\overline{K} \log \overline{K}} \right\} \right) \ll \frac{1}{\sqrt{\overline{K}}}. \end{aligned}$$

By the definition of $K_N(\alpha )$ and $\overline{K}$, this immediately gives

$$\begin{aligned} \nu \left( \left\{ \alpha \in [0,1] \,: \, |K_N(\alpha ) - \overline{K}| \ge C \sqrt{\overline{K} \log \overline{K}} \right\} \right) \ll \frac{1}{\sqrt{\overline{K}}}. \end{aligned}$$

Roughly speaking, this means that we can replace $K_N(\alpha )$ by $\overline{K}$ in the above formulas; the point is that the latter does not depend on $\alpha$. More precisely, outside a set of $\nu$-measure $\ll 1/\sqrt{\overline{K}}$, we have $D_2^2(S(\alpha , N)) = (1/360) \sum _{k=1}^{\overline{K}} a_k^2+Y_3$, where

$$\begin{aligned} |Y_3| \le \frac{C}{\overline{K}^{10}} \sum _{k=1}^{2 \overline{K}} a_k^2 + C \sum _{k=\overline{K}-C\sqrt{\overline{K} \log \overline{K}}}^{\overline{K} + C \sqrt{\overline{K} \log \overline{K}}} a_k^2 +C \sum _{k=1}^{2\overline{K}} a_k. \end{aligned}$$

Since $5 \pi ^3 /(\log N)^2 = 720 (\log 2)^2 /(\pi \overline{K}^2) +O(1/\overline{K}^3)$, normalizing the previous formula leads to the fact that outside a set of $\nu$-measure $\ll 1/\sqrt{\overline{K}}$,

$$\begin{aligned} 5 \pi ^3 \frac{D_2^2 (S(\alpha , N))}{(\log N)^2} = \frac{2 (\log 2)^2}{\pi \overline{K}^2} \sum _{k=1}^{\overline{K}} a_k^2 +Y_4, \end{aligned}$$

where

$$\begin{aligned} |Y_4| \le \frac{C}{\overline{K}^3} \sum _{k=1}^{2 \overline{K}} a_k^2 + \frac{C}{\overline{K}^2} \sum _{k=\overline{K}-C\sqrt{\overline{K} \log \overline{K}}}^{\overline{K} + C \sqrt{\overline{K} \log \overline{K}}} a_k^2 +\frac{C}{\overline{K}^2} \sum _{k=1}^{2\overline{K}} a_k. \end{aligned}$$

We now estimate the three error terms in the previous formula. The limit distribution with rate of Heinrich (11) gives

$$\begin{aligned} \nu \left( \left\{ \alpha \in [0,1] \,: \, \frac{1}{\overline{K}^3} \sum _{k=1}^{2 \overline{K}} a_k^2 \ge \frac{1}{\overline{K}^{1/3}} \right\} \right) \ll \int _{\textrm{const} \cdot \overline{K}^{2/3}}^{\infty } \frac{e^{-1/(2x)}}{\sqrt{2 \pi } x^{3/2}} \, \textrm{d} x + \frac{1}{\overline{K}^{1-\varepsilon }} \ll \frac{1}{\overline{K}^{1/3}}. \end{aligned}$$

Since the sequence $a_k$ is strictly stationary, we similarly deduce

$$\begin{aligned} \begin{aligned}&\nu \Bigg ( \Bigg \{ \alpha \in [0,1] \,: \, \frac{1}{\overline{K}^2} \sum _{k=\overline{K}-C \sqrt{\overline{K} \log \overline{K}}}^{\overline{K}+C \sqrt{\overline{K} \log \overline{K}}} a_k^2 \ge \frac{(\log \overline{K})^{1/3}}{\overline{K}^{1/3}} \Bigg \} \Bigg ) \\ {}&\quad = \nu \left( \left\{ \alpha \in [0,1] \,: \, \frac{1}{\overline{K}^2} \sum _{k=1}^{C \sqrt{\overline{K} \log \overline{K}}} a_k^2 \ge \frac{(\log \overline{K})^{1/3}}{\overline{K}^{1/3}} \right\} \right) \\ {}&\quad \ll \int _{\textrm{const} \cdot \overline{K}^{2/3}/(\log \overline{K})^{2/3}}^{\infty } \frac{e^{-1/(2x)}}{\sqrt{2 \pi } x^{3/2}} \, \textrm{d} x + \frac{1}{\overline{K}^{1/2-\varepsilon }} \\ {}&\quad \ll \frac{(\log \overline{K})^{1/3}}{\overline{K}^{1/3}}. \end{aligned} \end{aligned}$$

Finally, formula (12) gives

$$\begin{aligned} \nu \left( \left\{ \alpha \in [0,1] \,: \, \frac{1}{\overline{K}^2} \sum _{k=1}^{2\overline{K}} a_k \ge \frac{1}{\overline{K}^{1/3}} \right\} \right) \ll \frac{1}{\overline{K}^{2/3}}. \end{aligned}$$

By the previous three estimates, we can finally write

14 $$\begin{aligned} 5 \pi ^3 \frac{D_2^2 (S(\alpha , N))}{(\log N)^2} = \frac{2 (\log 2)^2}{\pi \overline{K}^2} \sum _{k=1}^{\overline{K}} a_k^2 +Y_5, \end{aligned}$$

where

15 $$\begin{aligned} \nu \left( \left\{ \alpha \in [0,1] \, : \, |Y_5| \ge C \frac{(\log \overline{K})^{1/3}}{\overline{K}^{1/3}} \right\} \right) \le C \frac{(\log \overline{K})^{1/3}}{\overline{K}^{1/3}} . \end{aligned}$$

The proof of the theorem is now immediate. Assume first, that $\mu$ is absolutely continuous with respect to the Lebesgue measure. The theorem of Samur (10) ensures that the main term in (14) converges in distribution to the standard Lévy distribution as *N*, and hence $\overline{K}$, goes to infinity. Since $Y_5 \rightarrow 0$ in $\nu$-measure, the same holds also in $\mu$-measure, and the convergence to the standard Lévy distribution remains true for the left hand side of (14). This finishes the proof for a general absolutely continuous measure $\mu$.

Next, let $\mu$ be either $\lambda$ or $\nu$. Then the sequence $a_k$ is $\psi$-mixing with exponential rate, and the limit distribution with rate of Heinrich (11) ensures that the main term in (14) converges to the standard Lévy distribution with rate $\ll 1/\overline{K}^{1-\varepsilon }$. The estimate (15), which holds also with $\lambda$ in place of $\nu$, together with the trivial fact that the distribution function of the Lévy distribution is Lipschitz, shows that this convergence remains true for the left hand side of (14) with the rate $\ll (\log \overline{K})^{1/3} / \overline{K}^{1/3}$. This finishes the proof of the rate of convergence for $\lambda$ and $\nu$. $\square$

## Typical rationals

Let $\mathcal {F}_Q$ denote the set of all reduced fractions in the interval (0, 1) with denominator at most *Q*, and let us write every $p/q \in \mathcal {F}_Q$ in the form $p/q=[0;a_1, \ldots , a_r]$. It does not matter which of the two possible expansions is chosen. Note that the partial quotients $a_1=a_1(p/q), \ldots , a_r=a_r(p/q)$ as well as the length $r=r(p/q)$ are functions of *p*/*q*. For the sake of simplicity, we use the convention $a_k=0$ if $k>r$.

The proof of Theorem 6 is based on recent results of Bettin and Drappeau on the limit distribution of power sums of the partial quotients; they are perfect analogues of the results for typical irrationals mentioned in Sect. 3.2.

### Lemma 11

(Bettin–Drappeau [4]) For any $Q \ge 2$ and $\varepsilon >0$,

16 $$\begin{aligned} \sup _{t \ge 0} \left| \frac{1}{|\mathcal {F}_Q|} \left| \left\{ \frac{p}{q} \in \mathcal {F}_Q \, : \, \frac{\pi ^3}{72 (\log Q)^2} \sum _{k=1}^r a_k^2 \le t \right\} \right| - \int _0^t \frac{e^{-1/(2x)}}{\sqrt{2 \pi } x^{3/2}} \, \textrm{d} x \right| \ll \frac{1}{(\log Q)^{1-\varepsilon }} \end{aligned}$$

and

$$\begin{aligned} \sup _{t \in \mathbb {R}} \left| \frac{1}{|\mathcal {F}_Q|} \left| \left\{ \frac{p}{q} \in \mathcal {F}_Q \,: \, \frac{1}{\log Q} \sum _{k=1}^r a_k - \frac{\log \log Q - \gamma }{\pi ^2/12} \le t \right\} \right| - G(t) \right| \ll \frac{1}{(\log Q)^{1-\varepsilon }} \end{aligned}$$

with implied constants depending only on $\varepsilon$. Here $\gamma$ is the Euler–Mascheroni constant, and *G*(*t*) is the distribution function of the law with characteristic function

$$\begin{aligned} \int _{\mathbb {R}} e^{itx} \, \textrm{d} G(t) = \exp \left( - \frac{6}{\pi } |x| \left( 1 + \frac{2i}{\pi } \textrm{sgn}(x) \log |x| \right) \right) . \end{aligned}$$

The second limit distribution in Lemma 11 immediately yields

17 $$\begin{aligned} \frac{1}{|\mathcal {F}_Q|} \left| \left\{ \frac{p}{q} \in \mathcal {F}_Q \, : \, \frac{1}{\log Q} \sum _{k=1}^r a_k \ge t + \frac{\log \log Q}{\pi ^2/12} \right\} \right| \ll \frac{1}{t} + \frac{1}{(\log Q)^{1-\varepsilon }} \quad \text {as } t \rightarrow \infty . \end{aligned}$$

Note that (16) was stated in [4] with the rate $\ll 1/(\log \log Q)^{1-\varepsilon }$, but the methods of that paper actually give $\ll 1/(\log Q)^{1-\varepsilon }$. For the sake of completeness, we deduce (16) as stated here in Sect. 4.1. We now prove a lemma which will serve as a substitute for the fact that the partial quotients are not exactly identically distributed, and then prove Theorem 6.

### Lemma 12

For any positive integers *Q*, *k*, *t*, we have

$$\begin{aligned} \left| \left\{ \frac{p}{q} \in \mathcal {F}_Q \,: \, a_k \ge t \right\} \right| \le \frac{2Q^2}{t}. \end{aligned}$$

### Proof

Assume first, that $k=1$. Note that $a_1 \ge t$ implies that $0 < p/q \le 1/t$. In particular, for each $1 \le q \le Q$ there are at most *q*/*t* possible numerators *p*, hence

18 $$\begin{aligned} \left| \left\{ \frac{p}{q} \in \mathcal {F}_Q \, : \, a_1 \ge t \right\} \right| \le \sum _{q=1}^Q \frac{q}{t} \le \frac{Q^2}{t} . \end{aligned}$$

Next, assume that $k \ge 2$. Let $\textrm{denom}(x)$ denote the denominator of a rational *x* (in its reduced form). From the recursion satisfied by the denominator of the convergents one readily deduces the supermultiplicative property

$$\begin{aligned} \textrm{denom}([0;a_1, \ldots , a_r]) \ge \textrm{denom}([0;a_1, \ldots ,a_{k-1}]) \cdot \textrm{denom}([0;a_k, \ldots , a_r]). \end{aligned}$$

For any fixed positive integers $b_1, \ldots , b_{k-1}$ we thus obtain

$$\begin{aligned}{} & {} \left| \left\{ \frac{p}{q} \in \mathcal {F}_Q \,: \, a_1=b_1, \ldots , a_{k-1}=b_{k-1}, \,\, a_k \ge t \right\} \right| \\ {}{} & {} \qquad \le \left| \left\{ \frac{p}{q} \in \mathcal {F}_{Q/\textrm{denom}([0;b_1, \ldots , b_{k-1}])} \,: \, a_1 \ge t \right\} \right| . \end{aligned}$$

Summing over $b_1, \ldots , b_{k-1}$ and applying (18) leads to

$$\begin{aligned} \left| \left\{ \frac{p}{q} \in \mathcal {F}_Q \,: \, a_k \ge t \right\} \right| \le \sum _{b_1, \ldots , b_{k-1}=1}^{\infty } \frac{Q^2}{t (\textrm{denom}([0;b_1, \dots , b_{k-1}]))^2}. \end{aligned}$$

Recall that the set of real numbers $[0;c_1, c_2, \ldots ] \in [0,1]$ such that $c_1=b_1, \ldots , c_{k-1}=b_{k-1}$ is an interval whose length is at least $1/(2 \, \textrm{denom}([0;b_1, \ldots , b_{k-1}])^2)$. Since these are pairwise disjoint intervals, we have

$$\begin{aligned} \sum _{b_1, \ldots , b_{k-1}=1}^{\infty } \frac{1}{(\textrm{denom}([0;b_1, \ldots , b_{k-1}]))^2} \le 2, \end{aligned}$$

and the claim follows. $\square$

### Proof of Theorem 6

Throughout the proof, $C>0$ is a large universal constant whose value changes from line to line, and $Z_i=Z_i (p/q)$, $i=1,2$ are error terms.

Proposition 7 and formula (4) show that we can write

$$\begin{aligned} D_2^2 (S(p/q,q)) = \frac{1}{360} \sum _{k=1}^r a_k^2 +Z_1, \quad \text {where} \quad |Z_1| \le C \sum _{k=1}^r a_k + \frac{C}{q} \sum _{k=0}^{r-1} a_{k+1}^3 q_k. \end{aligned}$$

Here $a_{k+1}^3 q_k \le a_{k+1}^2 q_{k+1}$, and $q_k/q=q_k/q_r \le 1/F_{r-k+1}$, where $F_k$ is the sequence of Fibonacci numbers. Hence normalizing the previous formula leads to

$$\begin{aligned}{} & {} 5 \pi ^3 \frac{D_2^2 (S(p/q,q))}{(\log Q)^2} = \frac{\pi ^3}{72 (\log Q)^2} \sum _{k=1}^r a_k^2 + Z_2, \quad \end{aligned}$$

where

$$\begin{aligned}{} & {} |Z_2| \le \frac{C}{(\log Q)^2} \sum _{k=1}^r a_k + \frac{C}{(\log Q)^2} \sum _{k=1}^r \frac{a_k^2}{F_{r-k+1}}. \end{aligned}$$

The first error term can be estimated in measure using formula (17) as

$$\begin{aligned} \frac{1}{|\mathcal {F}_Q|} \left| \left\{ \frac{p}{q} \in \mathcal {F}_Q \,: \, \frac{1}{(\log Q)^2} \sum _{k=1}^r a_k \ge \frac{1}{(\log Q)^{1/2}} \right\} \right| \ll \frac{1}{(\log Q)^{1/2}}. \end{aligned}$$

Let us agree for the moment to use the continued fraction expansion with $a_r \ge 2$ for fractions $p/q \in (0,1/2]$, and the expansion with $a_r=1$ for fractions $p/q \in (1/2,1)$. Then the map reversing the order of the partial quotients $\mathcal {F}_Q \rightarrow \mathcal {F}_Q$, $[0;a_1, a_2, \ldots , a_r] \mapsto [0; a_r, \ldots , a_2, a_1]$ is a bijection. In fact, $[0;a_r,\ldots , a_2,a_1]$ is the reduced fraction $q_{r-1}/q_r$, which has the same denominator as $[0;a_1,\ldots , a_r]$. Therefore the distribution of $(a_r, \ldots , a_2, a_1)$ is identical to that of $(a_1, a_2, \ldots , a_r)$, and we can apply Lemma 12 to estimate the second error term in measure as

$$\begin{aligned} \begin{aligned}&\frac{1}{|\mathcal {F}_Q|} \left| \left\{ \frac{p}{q} \in \mathcal {F}_Q \,: \, \frac{1}{(\log Q)^2} \sum _{k=1}^r \frac{a_k^2}{F_{r-k+1}} \ge \frac{1}{(\log Q)^{1/2}} \right\} \right| \\ {}&\quad = \frac{1}{|\mathcal {F}_Q|} \left| \left\{ \frac{p}{q} \in \mathcal {F}_Q \,: \, \sum _{k=1}^r \frac{a_k^2}{F_k} \ge (\log Q)^{3/2} \right\} \right| \\ {}&\quad \le \frac{1}{|\mathcal {F}_Q|} \sum _{k=1}^{\infty } \left| \left\{ \frac{p}{q} \in \mathcal {F}_Q \,: \, \frac{a_k^2}{F_k} \ge (\log Q)^{3/2} \right\} \right| \\ {}&\quad \le \frac{1}{|\mathcal {F}_Q|} \sum _{k=1}^{\infty } \frac{2Q^2}{F_k^{1/2} (\log Q)^{3/4}} \\ {}&\quad \ll \frac{1}{(\log Q)^{3/4}}. \end{aligned} \end{aligned}$$

Note that we used the convention $a_k=0$ if $k>r$, and the fact that $|\mathcal {F}_Q| \gg Q^2$. One readily checks that the value of $\sum _{k=1}^r a_k^2 / F_{r-k+1}$ for the two possible continued fraction expansions of the same rational differ at most by a factor of 2. Hence the tail estimate in the previous formula holds no matter which expansion we choose. In particular,

$$\begin{aligned} \frac{1}{|\mathcal {F}_Q|} \left| \left\{ \frac{p}{q} \in \mathcal {F}_Q \,: \, |Z_2| \ge \frac{1}{(\log Q)^{1/2}} \right\} \right| \ll \frac{1}{(\log Q)^{1/2}}, \end{aligned}$$

and the limit distribution theorem (16) of Bettin and Drappeau yields

$$\begin{aligned} \sup _{t \ge 0} \left| \frac{1}{|\mathcal {F}_Q|} \left| \left\{ \frac{p}{q} \in \mathcal {F}_Q \,: \, 5 \pi ^3 \frac{D_2^2 (S(p/q,q))}{(\log Q)^2} \le t \right\} \right| - \int _0^t \frac{e^{-1/(2x)}}{\sqrt{2 \pi } x^{3/2}} \, \textrm{d} x \right| \ll \frac{1}{(\log Q)^{1/2}}. \end{aligned}$$

The error of replacing $(\log Q)^2$ by $(\log q)^2$ is easily seen to be negligible compared to $1/(\log Q)^{1/2}$. $\square$

### Proof of Lemma 11

We now deduce the rate $\ll 1/(\log Q)^{1-\varepsilon }$ in (16). Fix $\varepsilon >0$. Applying the main result [4, Theorem 3.1] of Bettin and Drappeau to, in their notation, $\phi (x)=\lfloor 1/x \rfloor ^2$ with $\alpha _0=1/2-\varepsilon$, we conclude that there exist constants $t_0,\delta >0$ such that for all $|t| < t_0$,

19 $$\begin{aligned} \frac{1}{|\mathcal {F}_Q|} \sum _{p/q \in \mathcal {F}_Q} \exp \left( it \sum _{k=1}^r a_k^2 \right) = \exp \left( U(t) \log Q + O \left( |t|^{1/2-\varepsilon } + Q^{-\delta } \right) \right) , \end{aligned}$$

where

$$\begin{aligned} U(t) = \frac{12}{\pi ^2} \int _{0}^{1} \frac{e^{it\lfloor 1/x \rfloor ^2} -1}{1+x} \, \textrm{d}x + O \left( |t|^{1-\varepsilon } \right) = \frac{12}{\pi ^2} \int _{1}^{\infty } \frac{e^{it \lfloor x \rfloor ^2}-1}{x^2+x} \, \textrm{d}x + O \left( |t|^{1-\varepsilon } \right) . \end{aligned}$$

Here $t_0, \delta$ and the implied constants depend only on $\varepsilon$.

Our improvement in (16) comes from a more careful estimate for *U*(*t*). Assume that $0<t<t_0$. Since $|\lfloor x \rfloor ^2 -x^2| \le 2x$, the error of removing the integer part function is negligible:

$$\begin{aligned} \left| \int _{1}^{\infty } \frac{e^{i t \lfloor x \rfloor ^2} - e^{i t x^2}}{x^2+x} \, \textrm{d}x \right| \le \int _{1}^{\infty } \frac{\min \{ 2 t x, 2 \}}{x^2+x} \, \textrm{d} x \ll t \log \frac{1}{t}. \end{aligned}$$

Therefore

$$\begin{aligned} U(t) = \frac{12}{\pi ^2} \int _{1}^{\infty } \frac{e^{itx^2}-1}{x^2+x} \, \textrm{d} x +O(t^{1-\varepsilon }) = \frac{12 \sqrt{t}}{\pi ^2} \int _{\sqrt{t}}^{\infty } \frac{e^{ix^2}-1}{x^2+\sqrt{t}x} \, \textrm{d} x +O(t^{1-\varepsilon }). \end{aligned}$$

We now compare the remaining integral to its limit, the Fresnel-type integral $\int _{0}^{\infty } (e^{ix^2}-1)/x^2 \, \textrm{d} x = (i-1) \sqrt{2 \pi }/2$. We have

$$\begin{aligned} \begin{aligned} \left| \int _{\sqrt{t}}^{\infty } \frac{e^{ix^2}-1}{x^2+\sqrt{t}x} \, \textrm{d} x - \int _{0}^{\infty } \frac{e^{ix^2}-1}{x^2} \, \textrm{d} x \right|&\le \left| \int _{0}^{\sqrt{t}} \frac{e^{ix^2}-1}{x^2} \, \textrm{d} x \right| \\&\quad + \int _{\sqrt{t}}^{\infty } |e^{ix^2}-1| \cdot \left| \frac{1}{x^2+\sqrt{t}x} - \frac{1}{x^2} \right| \, \textrm{d} x \\&\le \int _{0}^{\sqrt{t}} 1 \, \textrm{d} x + \int _{\sqrt{t}}^{\infty } \min \{ x^2, 2 \} \frac{\sqrt{t}}{x^3} \, \textrm{d} x \\ {}&\ll \sqrt{t} \log \frac{1}{t}, \end{aligned} \end{aligned}$$

hence $U(t)= \frac{6 \sqrt{2} \sqrt{t}}{\pi ^{3/2}} (i-1) + O(t^{1-\varepsilon })$. The case of negative *t* follows from complex conjugation, thus for $|t|<t_0$,

20 $$\begin{aligned} U(t) = - \frac{6 \sqrt{2} |t|^{1/2}}{\pi ^{3/2}} (1- i \textrm{sgn}(t)) + O(|t|^{1-\varepsilon }) . \end{aligned}$$

Now let

$$\begin{aligned} \varphi _1(t)= \frac{1}{|\mathcal {F}_Q|} \sum _{p/q \in \mathcal {F}_Q} \exp \left( i t \frac{\pi ^3}{72 (\log Q)^2} \sum _{k=1}^r a_k^2 \right) \end{aligned}$$

and $\varphi _2(t)=\exp (-|t|^{1/2}(1-i \textrm{sgn}(t)))$; the latter is the characteristic function of the standard Lévy distribution. The Berry–Esseen inequality [24, p. 142] states that the distance of these two distributions in the Kolmogorov metric is, with any $T>0$,

$$\begin{aligned}{} & {} \sup _{t \ge 0} \left| \frac{1}{|\mathcal {F}_Q|} \left| \left\{ \frac{p}{q} \in \mathcal {F}_Q \,: \, \frac{\pi ^3}{72 (\log Q)^2} \sum _{k=1}^r a_k^2 \le t \right\} \right| - \int _0^t \frac{e^{-1/(2x)}}{\sqrt{2 \pi } x^{3/2}} \, \textrm{d} x \right| \\ {}{} & {} \qquad \ll \frac{1}{T} + \int _{0}^{T} \frac{|\varphi _1(t)-\varphi _2(t)|}{t} \, \textrm{d} t. \end{aligned}$$

Choose $T = \log Q$. Formulas (19) and (20) show that for $|t| \le \log Q$,

$$\begin{aligned} \begin{aligned} \varphi _1(t)&= \varphi _2(t) \exp \left( O \left( \left( \frac{|t|}{(\log Q)^2} \right) ^{1-\varepsilon } \log Q + \left( \frac{|t|}{(\log Q)^2} \right) ^{1/2-\varepsilon } + Q^{-\delta } \right) \right) \\ {}&= \varphi _2(t) \left( 1 + O \left( \frac{|t|^{1-\varepsilon }+|t|^{1/2-\varepsilon }}{(\log Q)^{1-2 \varepsilon }} + Q^{-\delta } \right) \right) . \end{aligned} \end{aligned}$$

Using $|\varphi _2(t)|= e^{-|t|^{1/2}}$, this immediately yields

$$\begin{aligned} |\varphi _1(t)-\varphi _2(t)| \ll e^{-|t|^{1/2}} \left( \frac{|t|^{1-\varepsilon }+|t|^{1/2-\varepsilon }}{(\log Q)^{1-2 \varepsilon }} + Q^{-\delta } \right) . \end{aligned}$$

It is now easy to see that

$$\begin{aligned} \int _{Q^{-100}}^{1} \frac{|\varphi _1(t)-\varphi _2(t)|}{t} \, \textrm{d} t \ll \frac{1}{(\log Q)^{1-2 \varepsilon }} \quad \text {and} \quad \int _{1}^{\log Q} \frac{|\varphi _1(t)-\varphi _2(t)|}{t} \, \textrm{d} t \ll \frac{1}{(\log Q)^{1-2 \varepsilon }}. \end{aligned}$$

On the other hand, by a very rough estimate we have $\sum _{k=1}^r a_k^2 \le Q^3$, hence $|\varphi _1(t)-1| \ll |t| Q^3$. Clearly $|\varphi _2(t)-1| \ll |t|^{1/2}$, thus

$$\begin{aligned} \int _{0}^{Q^{-100}} \frac{|\varphi _1(t)-\varphi _2(t)|}{t} \, \textrm{d} t \ll \int _{0}^{Q^{-100}} \frac{t Q^3 + t^{1/2}}{t} \, \textrm{d} t \ll Q^{-50}. \end{aligned}$$

Therefore

$$\begin{aligned} \frac{1}{\log Q} + \int _{0}^{\log Q} \frac{|\varphi _1(t)-\varphi _2(t)|}{t} \, \textrm{d} t \ll \frac{1}{(\log Q)^{1-2 \varepsilon }}, \end{aligned}$$

as claimed.

## Footnotes

1 Both $\Gamma$ and its dual have full rank *d*. The terminology “rank-1” comes from the fact that the set $\mathcal {L}_{p_1, \ldots , p_d,N}$, viewed as a finite additive subgroup of the torus $\mathbb {R}^d / \mathbb {Z}^d$, is cyclic.

2 If $a_1=1$, then the term $k=0$ can be removed.

## References

[1] Beck, J.: Probabilistic Diophantine Approximation. Randomness in Lattice Point Counting. Springer Monographs in Mathematics, Springer, Cham (2014)

[2] Beck, J.: Randomness of the square root of 2 and the giant leap, part 1. Period. Math. Hungar. **60**, 137–242 (2010) 10.1007/s10998-010-2137-9

[3] Beck, J.: Randomness of the square root of 2 and the giant leap, part 2. Period. Math. Hungar. **62**, 127–246 (2011) 10.1007/s10998-011-6127-3

[4] Bettin, S., Drappeau, S.: Limit laws for rational continued fractions and value distribution of quantum modular forms. Proc. Lond. Math. Soc. **125**, 1377–1425 (2022) 10.1112/plms.12485

[5] Bilyk, D.: The discrepancy of irrational lattices. In: Monte Carlo and quasi-Monte Carlo methods 2012, 289–296, Springer Proc. Math. Stat., 65, Springer, Heidelberg (2013)

[6] Bilyk, D., Temlyakov, V., Yu, R.: Fibonacci sets and symmetrization in discrepancy theory. J. Complexity **28**, 18–36 (2012) 10.1016/j.jco.2011.07.001

[7] Bilyk, D., Temlyakov, V., and Yu, R.: The discrepancy of two-dimensional lattices. Recent advances in harmonic analysis and applications, 63–77, Springer Proc. Math. Stat., 25, Springer, New York (2013)

[8] Borda, B.: On the distribution of Sudler products and Birkhoff sums for the irrational rotation. To appear in Ann. Inst. Fourier (Grenoble)

[9] Borda, B.: On the theorem of Davenport and generalized Dedekind sums. J. Number Theory **172**, 1–20 (2017) 10.1016/j.jnt.2016.08.016

[10] Borda, B.: The number of lattice points in irrational polytopes. Ph.D. Thesis, Rutgers The State University of New Jersey—New Brunswick. ProQuest LLC (2016)

[11] Bykovskii, V. A.: The discrepancy of the Korobov lattice points. (Russian) Izv. Ross. Akad. Nauk Ser. Mat. 76: 19–38. English translation in Izv. Math. **76**(2012), 446–465 (2012)

[12] Davenport, H.: Note on irregularities of distribution. Mathematika **3**, 131–135 (1956) 10.1112/S0025579300001807

[13] Diamond, H., Vaaler, J.: Estimates for partial sums of continued fraction partial quotients. Pac. J. Math. **122**, 73–82 (1986) 10.2140/pjm.1986.122.73

[14] Dick, J., Kritzer, P., and Pillichshammer, F.: Lattice Rules—Numerical Integration, Approximation, and Discrepancy. With an appendix by Adrian Ebert. Springer Series in Computational Mathematics, 58. Springer, Cham (2022)

[15] Dick, J., Pillichshammer, F.: Digital Nets and Sequences. Discrepancy Theory and Quasi-Monte Carlo Integration, Cambridge University Press, Cambridge (2010)

[16] Drmota, M., Tichy, R.: Sequences, Discrepancies and Applications. Lecture Notes in Mathematics, vol. 1651. Springer-Verlag, Berlin (1997)

[17] Heinrich, L.: Rates of convergence in stable limit theorems for sums of exponentially -mixing random variables with an application to metric theory of continued fractions. Math. Nachr. **131**, 149–165 (1987) 10.1002/mana.19871310114

[18] Hinrichs, A., Kritzinger, R., Pillichshammer, F.: Extreme and periodic discrepancy of plane point sets. Acta Arith. **199**, 163–198 (2021) 10.4064/aa200520-22-12

[19] Illarionov, A. A.: A probability estimate for the discrepancy of Korobov lattice points. (Russian) Mat. Sb. 212,: 73–88. English translation in Sb. Math. **212**(2021), 1571–1587 (2021)

[20] Illarionov, A. A. : Distribution of Korobov-Hlawka sequences. (Russian) Mat. Sb. 213,: 70–96. English translation in Sb. Math. **213**(2022), 1222–1249 (2022)

[21] Iosifescu, M., Kraaikamp, C.: Metrical Theory of Continued Fractions. Mathematics and its Applications, vol. 547. Kluwer Academic Publishers, Dordrecht (2002)

[22] Kesten, H.: The discrepancy of random sequences . Acta Arith. **10**, 183–213 (1964) 10.4064/aa-10-2-183-213

[23] Morita, T.: Local limit theorem and distribution of periodic orbits of Lasota-Yorke transformations with infinite Markov partition. J. Math. Soc. Jpn. **46**, 309–343 (1994) 10.2969/jmsj/04620309

[24] Petrov, V.: Limit Theorems of Probability Theory. Sequences of Independent Random Variables. Oxford Studies in Probability, 4. Oxford Science Publications. The Clarendon Press, Oxford University Press, New York (1995)

[25] Roçadas, L., Schoißengeier, J.: An explicit formula for the -discrepancy of -sequences. Computing **77**, 113–128 (2006) 10.1007/s00607-005-0143-1

[26] Roth, K.: On irregularities of distribution. Mathematika **1**, 73–79 (1954) 10.1112/S0025579300000541

[27] Samur, J.: On some limit theorems for continued fractions. Trans. Am. Math. Soc. **316**, 53–79 (1989) 10.1090/S0002-9947-1989-0948197-9

[28] Schoissengeier, J.: Another proof of a theorem of J. Beck. Monatsh. Math. **129**, 147–151 (2000) 10.1007/s006050050014

[29] Takahasi, H.: Large deviations for denominators of continued fractions. Nonlinearity **33**, 5861–5874 (2020) 10.1088/1361-6544/ab9a1d

